# From Seeds to Cell: Improving PEMFC Performance and Durability by Seed‐Mediation Synthesis for PtNiIr ORR Nanocatalysts

**DOI:** 10.1002/advs.202505958

**Published:** 2025-05-20

**Authors:** Lujin Pan, Thomas Merzdorf, Carlos A. Campos‐Roldàn, An Guo, Jiasheng Lu, Johannes Schmidt, Marc Heggen, Malte Klingenhof, Xingli Wang, Sebastian Möhle, Sören Selve, Deborah Jones, Peter Strasser

**Affiliations:** ^1^ Department of Chemistry Technische Universität Berlin Strasse des 17. Juni 124 10623 Berlin Germany; ^2^ ICGM CNRS ENSCM Univ. Montpellier Cedex 5 Montpellier 34095 France; ^3^ Ernst Ruska‐Centre for Microscopy and Spectroscopy with Electrons Forschungszentrum Juelich GmbH 52425 Juelich Germany; ^4^ Center for Electron Microscopy (ZELMI) Technische Universität Berlin Strasse des 17. Juni 135 10623 Berlin Germany

**Keywords:** durability, electrochemistry, fuel cells, seed‐mediation synthesis, stability

## Abstract

Proton exchange membrane fuel cells (PEMFCs) provide efficient, green power solutions. However, the sluggish kinetics of the oxygen reduction reaction (ORR) at the cathode, with its need for elevated Pt loadings, lowers efficiency and raises cost, which hinders their wider implementation. Pt‐based designer alloy electrocatalysts, more specifically ternary PtNiX nanocatalysts, hold great potential for improving ORR activity and thus overall cell performance. This study explores synthesis and performance evaluations of novel ternary PtNiIr ORR catalysts prepared using seed‐mediation at different catalyst loadings and deposited on various carbon support materials. Membrane electrode assembly (MEA) performance evaluations are carried out to assess catalyst activity and stability under operating conditions, revealing better performance and durability for seed‐mediated catalysts compared to the non‐seed‐mediated catalyst used as a reference. The results showed further improved performance and durability of the seed‐mediated catalysts on porous carbon than solid carbon, due to the deposition of catalyst nanoparticles inside the carbon pores. Degradation analysis using online inductively coupled plasma – mass spectrometry (ICP‐MS) indicated the dissolution of metals during contact with the electrolyte and under operating conditions, confirming the observed catalyst stability trends in MEA. The experiments highlighted the impact of catalyst composition and supports on the stability of the materials.

## Graphical abstract

The seed‐mediation synthesis approach offers increased activity and stability for the PtNiIr nanocatalysts. It is designed as an oxygen reduction reaction catalyst in proton exchange membrane fuel cells (PEMFC), further enhanced by deposing the particles inside the porous carbon support material. The improved stability of the particles is confirmed by *online* inductively coupled plasma mass spectrometry (ICP‐MS) measurements.

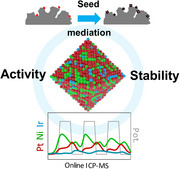

## 1 Introduction

Proton exchange membrane fuel cells (PEMFCs) have emerged as a promising technology for clean energy conversion due to their high efficiency and zero carbon emissions compared to conventional combustion engines.^[^ ^1^ ^]^ A critical challenge in optimizing PEMFC performance lies in the cathode due to the sluggish kinetics, where the oxygen reduction reaction (ORR) takes place.^[^ ^2^ ^]^ Consequently, it is crucial to develop active and durable cathode catalysts for the PEMFC advancement. While platinum‐based catalysts, especially PtNi alloys, have achieved the state‐of‐the‐art ORR performance, there is a growing focus toward structurally sophisticated and more active catalysts, and these advanced catalysts offer unique surface geometries and improved intrinsic activities, which are critical for enhancing ORR efficiency.^[^ ^3^ ^]^ While these catalysts have demonstrated high activity in laboratory rotating disk electrode (RDE) testing, their performance in PEMFC membrane electrode assemblies (MEA) has been less effective, highlighting a critical need for innovative approaches to fully realize their potential in practical applications.^[^ ^4^ ^]^

A promising strategy is the seed‐mediated synthesis approach, which offers a more controlled approach toward catalyst nanoparticles with desired properties such as a high Pt loading of up to 40 wt% while maintaining their high catalytic activity. Recent studies have shown that using Ir‐based electrocatalyst can increase the ORR electrochemical performance.^[^ ^5^ ^]^ Strickler et al. also discovered the stability enhancement effect of Ir@Pt core‐shell catalysts for ORR.^[^ ^6^ ^]^ This concept could be incorporated into our system to verify the stabilization effect mechanism in Pt‐based ternary alloys. Moreover, studies have shown that increasing the accessible mesoporous structure within carbon supports can significantly improve the performance of PEMFC cathodes by facilitating better oxygen diffusion and reactant accessibility.^[^ ^7^ ^]^ For example, incorporating highly porous carbon in the PtNi alloy catalysts has been shown to enhance ORR activity by optimizing the distribution and accessibility of catalytic sites, as well as improving the durability of the catalyst under operational conditions.^[^ ^7a^ ^]^

This study investigates the synthesis–structure–performance relationships of seed‐mediated PtNiIr catalysts, with a focus on three key aspects: i) the role of Ir as a dopant and seed material in controlling nanoparticle morphology and stability, ii) the Pt weight loading effects and iii) the influence of carbon support selection (solid versus porous) on ORR activity and durability. To evaluate this, RDE and MEA measurements were conducted. Additionally, *online* inductively coupled plasma mass spectrometry (ICP‐MS) measurements were performed to understand how the catalysts are dissolved during electrochemical testing.

In the end, this study reveals synthesis–structure–performance relationships of seed‐mediated shaped Pt alloy ORR catalysts and yields a deeper understanding of their material properties. By examining the influence of synthesis parameters and the role of different seeds, we offer insights that could lead the way for more robust and efficient catalysts for PEMFC cathodes.

## 2 Results and Discussion

A series of carbon‐supported ternary PtNiIr nanoparticle catalysts were prepared using a solvothermal, autoclave‐based synthesis method, incorporating a seed‐mediated approach to control particle nucleation and growth (Figure S1, Supporting Information). For the seed‐mediated synthesis, Pt or Ir precursor salts were initially reduced to metal seeds located on carbon materials (i.e., Pt/C or Ir/C) (Figure S2, Supporting Information). Subsequently, these nanoparticles served as nucleation seeds for the solvothermal reactive nucleation and growth of the ternary PtNiIr catalyst particles. To evaluate the effect of seed‐mediation, a non‐seed‐mediated ternary PtNiIr reference catalyst was prepared under identical conditions. The catalyst material samples are henceforth labeled as “XS10‐LY‐C_Z_”: Here, “X” represents the type of seed metal, either “Pt”, “Ir”, or “N” for the non‐seed‐mediated sample, the number following S indicates the Pt or Ir wt% metal loading after the first synthesis step, Y refers to the Pt wt% loading (L) of the final catalyst and “Z” represents the nature of the carbon support, either “V” for Vulcan (Vulcan XC72R) or “KB” for KB300 (Ketjenblack EC‐300J), representing a solid and a porous carbon, respectively. The compositions of the different catalysts were determined by ICP‐OES analysis and are shown in Table S1 (Supporting Information). All three Pt seed‐mediated samples showed a Pt/Ni molar ratio of 2.3 to 2.8, which was obviously higher compared to the non‐seed‐mediated and the Ir seed‐mediated catalysts. All samples, except for the Ir‐seed‐mediated catalyst have an Ir content of less than 2 at%, which was lower than the nominal ratio of the initial precursors amount (5 at%). The Ir seed‐mediated sample (IrS10‐L30‐C_V_) had a significantly higher amount of Ir with 9.3 at% to assess if the enhanced stability effect of high amounts of Ir is also observable for our oh‐PtNi nanoparticle catalysts.

**Figure 1:**
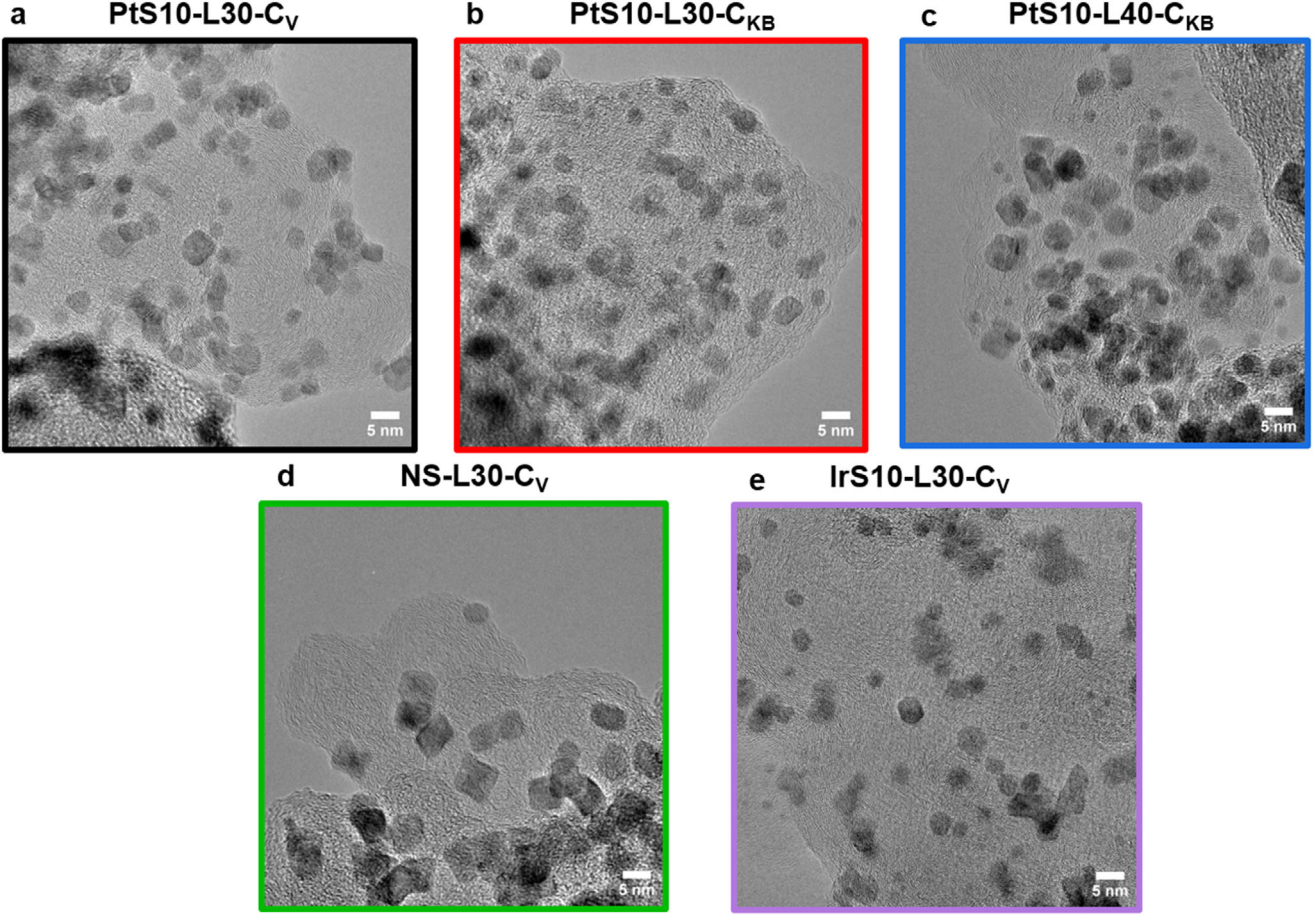
TEM images of ternary PtNiIr nanoparticle electrocatalysts, prepared with seed‐mediation “PtS/IrS” or without seed mediation “NS”: a) PtS10‐L30‐C_V_; b) PtS10‐L30‐C_KB_; c) PtS10‐L40‐CK_B_; d) NS‐L30‐C_V_; e) IrS10‐L30‐C_V_; the scale bars in the images are 5 nm.

**Figure** **1** displays transmission electron microscopy (TEM) images of the five different carbon‐supported alloy nanoparticle catalysts, with the particle size distribution shown in Figure S3 (Supporting Information). The seed‐mediated samples exhibit various nanoparticle morphologies, including both spherical and octahedral shapes with particle sizes between 4 and 5 nm. PtS10‐L30‐C_V_ had slightly bigger particles of 4.8 nm, compared to the other two seed‐mediated catalysts with 30 wt% loading (PtS10‐L30‐C_KB_ and IrS10‐L30‐C_V_) with slightly below 4 nm average sizes. PtS10‐L40‐C_KB_ has, with 4.4 nm, larger particles than PtS10‐L30‐C_KB_. All four seed‐mediated catalysts exhibit a slight asymmetric distribution with a higher abundance of bigger particles. In contrast, the non‐seed‐mediated samples, NS‐L30‐C_V_ exhibited larger particles with an average size of 6.6 nm, a result of the synthetic procedure, and these particles displayed a clear octahedral morphology.

Figure S4 (Supporting Information) shows scanning transmission electron microscopy (STEM) results, combining secondary electron (SE), annular dark‐field (DF), and bright‐field (BF) STEM images of PtS10‐L30‐C_V_ and PtS10‐L30‐C_KB_. The SE‐STEM images revealed the surface morphology of the oh‐PtNiIr as well as the carbon support, while the BF‐ and DF‐STEM images provided information on all nanoparticles situated both at the carbon surface, inside carbon pores, and at the carbon back side. Comparing these images indicated that parts of the catalyst particles in PtS10‐L30‐C_KB_ are located inside the pores, whereas those on Vulcan were not, this phenomenon is extensively reported in previous studies.^[^ ^8^ ^]^

The XRD patterns of five PtNiIr samples (Figure S5, Supporting Information) were displayed to investigated the alloy and crystal structures. The diffraction peaks observed at 2*θ* values of 40.6°, 47.4°, 69°, and 83.2° correspond to the (111), (200), (220), and (311) crystallographic planes, respectively, of a face‐centered cubic (fcc) structure. These peaks are characteristic of Pt‐based alloys and confirm the formation of a crystalline PtNiIr phase in all samples. Additionally, a peak observed at ≈25° is attributed to the (002) plane of graphitic carbon, indicating the presence of a carbon support material.

The near‐surface composition and electronic structure of all samples were investigated using X‐ray photoelectron spectroscopy (XPS). As shown in Figure S6 (Supporting Information), the five samples exhibited similar trends in the Pt 4f spectrum. Two prominent peaks at 71.3 and 74.7 eV correspond to metallic Pt 4f_7/2_ and Pt 4f_5/2_, respectively, indicating mostly metallic Pt in the particles. The Ni 2p XPS spectra (Figure S7, Supporting Information) revealed peaks indicative of higher valent Ni^2+^ and lower valent metallic Ni^0^ states, with varying intensities across the samples. All samples displayed peaks at 852.8 and 870.0 eV, indicating the existence of metallic Ni near the surface. Particularly, the PtS10‐L30‐C_KB_ sample showed the highest integrated peak intensity of Ni^2+^ compared to Ni^0^, while the IrS10‐L30‐C_V_ sample showed the lowest Ni^2+^ intensity. The differences in Ni^2+^ intensity among the catalysts could be due to various factors, such as incomplete reduction processes during synthesis, and the influence of carbon porosity, as well as the catalyst loading on nickel's oxidation states. Due to the low Ir content in all Pt seed‐mediated samples and the strong interference with the Pt 4f signal, only the IrS10‐L30‐C_V_ sample showed pronounced Ir 4f peaks, which indicate Iridium in the metallic state (Figure S8, Supporting Information).

**Figure 2:**
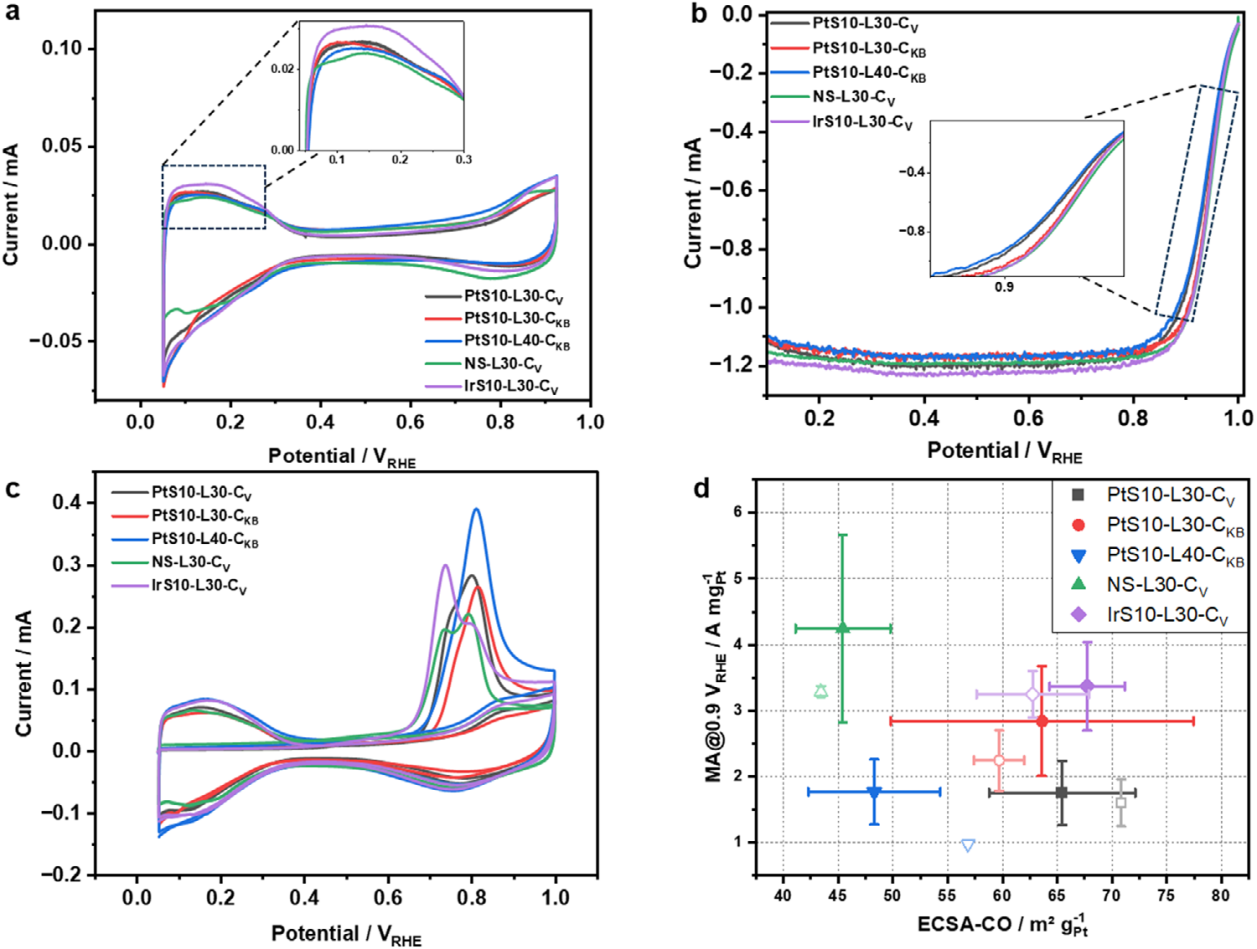
RDE results of S10‐L30‐C_V_ (black), S10‐L30‐C_KB_ (red), S10‐L40‐C_KB_ (blue), NS‐L30‐C_V_ (green), IrS10‐L30‐C_V_ (violet); a) cyclic voltammetry (CV), scanning rate 20 mV s^−1^. from 0.05 to 0.925 V_RHE_, inset is a zoom‐in on the H_upd_ region; b) linear sweep voltammetry (LSV), scanning rate 20 mV s^−1^, from 0.05 to 1.0 V_RHE_, RDE measured in 0.1 M HClO_4_; c) cyclic voltammetry of CO striping, with a scanning rate of 20 mV s^−1^, from 0.05 to 1.0 V_RHE_; d) Mass activity at 0.9 V_RHE_ as a function of ECSA‐CO before and after AST. Filled and hollow symbols represent the performance before (BOL) and after (EOL) AST.

The catalytic activity of all samples was pre‐screened using RDE measurements in acidic media (0.1 M HClO_4_). The alloys were applied as powder catalyst thin‐film without pre‐leaching. **Figure** **2**a shows the cyclic voltammetry (CV). The profiles reveal distinct electrochemical regions: hydrogen adsorption/desorption between 0.05 and 0.35 V_RHE_, the double‐layer region between 0.35 and 0.6 V_RHE_, and OH adsorption/desorption above 0.6 V_RHE_. These features confirm the presence of a Pt‐rich surface layer, as the characteristic peaks and regions align with those typically observed for Pt‐based materials. In the inset of Figure 2a, the curves demonstrate typical redox charge features of the Pt‐based (111) surfaces in the potential region lower than 0.3 V_RHE_.^[^ ^3e^ ^]^ For the non‐seed‐mediated catalyst, the hydroxyl adsorption/desorption peak shifted to lower potentials, suggesting a somewhat stronger surface hydroxyl adsorption.^[^ ^9^ ^]^ The corresponding linear sweep voltammetry (LSV) in an O_2_‐saturated electrolyte is displayed in Figure 2b. The ORR polarization curves show only slight differences in their diffusion‐limited current and their onset potential. The mixed‐control (kinetic and mass transport, shown in the inset) current region of NS‐L30‐C_V_, IrS10‐L30‐C_V_, and PtS10‐L30‐C_KB_ shifted toward more anodic potentials compared to the other two samples. Figure 2c shows the CO stripping CV used to determine the electrochemical surface area (ECSA‐CO). The trends in Pt‐based mass activity (MA) at 0.9 V_RHE_ is plotted against the ECSA‐CO in Figure 2d. As expected, one can discern a general positive correlation between initial MA and ECSA. There are some outliners, however: while the experimental ECSA value of NS‐L30‐C_V_ of 45 m^2^ g_Pt_^−1^ was the lowest, consistent with its largest TEM particle size, its MA value of over 4 A mg_Pt_^−1^ ranged highest among the catalysts; this could be due to more dominant octahedral‐shaped nanoparticles shown in this sample. By contrast, all seed‐mediated samples with a nominal Pt loading of 30 wt% achieved higher ECSAs of ≈65 m^2^ g_Pt_^−^1, compared to NS‐L30‐C_V_. The enhanced ECSA is attributed to smaller alloy particles resulting from the seed‐mediated synthesis method, as shown in the TEM images.

To evaluate the catalyst stability in the idealized RDE cell, accelerated stress tests (ASTs), were carried out, consisting of 10 k cycles from 0.6 to 0.925 V_RHE_ with a scanning rate of 100 mV s^−1^ in N_2_ atmosphere (CVs and LSVs before and after the AST are shown in Figure S9, Supporting Information). It was evident that both seed‐mediated on Vulcan‐supported catalysts displayed much more favorable performance stability over the KB‐supported or non‐seed‐mediated catalysts. The PtS10‐L30‐C_V_ catalyst exhibited stable performance near 2 A mg_Pt_^−1^ with minimal activity loss after AST, indicating high stability. The average ECSA‐CO increased after the AST within the error. PtS10‐L30‐C_KB_ showed high initial activity of 3 A mg_Pt_^−1^, while PtS10‐L40‐C_KB_ showed lower initial performance near 2 A mg_Pt_^−1^ that further declined after the stability test; its ECSA‐CO increased sharply after the AST to more than 55 m^2^ g_Pt_^−1^. This could be explained by a possible catalyst surface reconstruction exposing more surface area at the cost of stability. NS‐L30‐C_V_ exhibited the highest initial catalytic ORR activity among all 5 catalysts; however, it underwent a noticeable performance decline during the stability test. In contrast, the Ir seed‐mediated catalyst, IrS10‐L30‐C_V_, displayed excellent initial mass activity of over 3 A mg_Pt_^−1^ with only a minor performance decrease after the AST, suggesting favorable stability. The minimal change in its ECSA‐CO further highlights the catalyst's sustained activity in liquid media.

**Figure 3:**
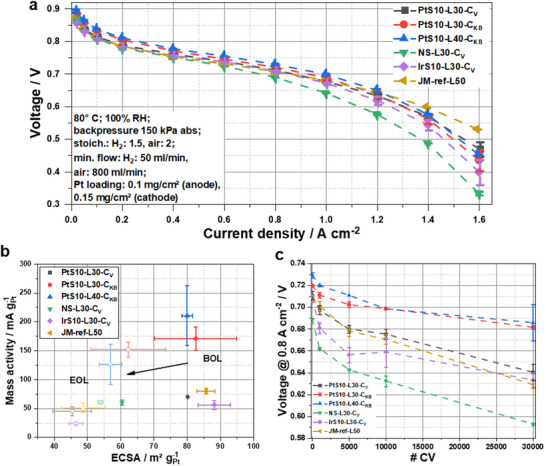
MEA results of S10‐L30‐C_V_ (black), S10‐L30‐C_KB_ (red), S10‐L40‐C_KB_ (blue), NS‐L30‐C_V_ (green), IrS10‐L30‐C_V_ (violet), JM 50 wt%Pt (gold); a) BOL polarization curve of 10 cm^2^ MEAs; b) mass activity (MA) and ECSA before and after AST; c) voltage evolution at 0.8 A cm^2^ during catalyst degradation AST.

After the RDE tests, single‐cell fuel cell investigations of the beginning‐of‐life (BOL) performance of the seed‐mediated cathode catalysts in more realistic environments were conducted. The present PEMFC tests are by no means comprehensive, not including detailed humidity or temperature studies. They were meant for an initial evaluation of performance trends under standard PEMFC conditions. MEAs were prepared for each catalyst following published procedures.^[^ ^10^ ^]^ After the break‐in procedure, BOL polarization curves were performed and are depicted in **Figure** **3**a. A Johnson Matthey (JM) 50 wt% Pt/C reference catalyst was tested as well. The advantages of the porous carbon support material are evident at low current (< 0.4 A cm^−2^) densities: the ternary Pt alloy catalysts supported on solid Vulcan, as well as the JM reference, revealed lower cell voltages, whereas both porous KB300‐based ORR catalysts showed significantly higher cell voltages. The catalyst nanoparticles embedded into the large number of (meso)pores (2–50 nm) of the KB300‐supported catalysts appear to boost the intrinsic activity of these catalysts.^[^ ^7^, ^11^ ^]^ Unlike support porosity, seed mediation does not appear to unfold a significant performance advantage in this regime.

At higher current densities, a different behavior emerged. Now, seed‐mediated catalysts performed significantly better than the non‐seed‐mediated catalysts. Both KB300‐supported seed‐mediated catalysts showed a larger undesired voltage drop compared to PtS10‐L30‐C_V_ and the JM reference, as expected due to larger oxygen transport limitations and resistances in the pores. Clearly, the seed‐mediated catalysts outperformed the non‐seed‐mediated catalyst (NS‐L30‐C_V_) at intermediate and high current densities (> 0.6 A cm^2^), demonstrating the performance improvement of the seed‐mediated synthesis method. Comparing Ir seed‐mediated and Pt seed‐mediated catalysts, significant performance differences were only observed at high current densities, with the Ir‐seed‐based materials showing a slightly larger voltage drop. The MA was also measured during PEMFC testing (Figure 3b), revealing that the three Vulcan‐based catalysts and the JM reference showed comparable MAs between 60 and 80 mA g_Pt_^−1^, while both catalysts supported on KB300 exhibited higher MAs of over 170 mA g_Pt_^−1^. These results are in agreement with the literature, indicating that the porous KB300 support with its accessible alloy particles on the surface and in pores under the given humidity conditions enhances the intrinsic activity of the catalyst, resulting in higher MA and higher onset potential in the MEA.^[^ ^12^ ^]^ The four seed‐mediated catalysts and the JM reference showed comparable ECSAs between 80 and 90 m^2^ g_Pt_^−1^, while the non‐seed‐mediated sample had a lower ECSA due to the bigger particle size. The ECSA was also measured at lower relative humidity (RH) (Figure S10, Supporting Information). At 30 % RH, the Vulcan‐based seed‐mediated catalysts and the JM reference exhibited higher ECSAs compared to the KB300‐based catalysts. This result is reasonable, since the particles of these latter catalysts were located to a larger extent inside the pores, where the proton accessibility drops faster under dry conditions.^[^ ^7b^ ^]^ Comparing the activity trends of RDE and MEA measurements, one must state that RDE‐based mass activity trends revealed only a moderate predictive power for the BOL catalytic ORR performance of single cell PEMFC under the chosen conditions (Figure 3b).

An AST was performed to investigate the cell and catalyst durability, consisting of 30000 square wave voltammograms between 0.6 and 0.95 V with a 3 s dwell time. Throughout the AST, polarization curves were measured to study the performance changes of the different catalysts. Figure S11 (Supporting Information) shows the first (BOL) and last (EOL, end‐of‐life) polarization curves of each catalyst. After the AST, both KB300‐based catalysts exhibit higher cell voltages at all current densities, underlining the improved durability of the seed‐mediated catalysts on porous support materials. The cell voltage at 0.8 A cm^−^^2^ over the course of the AST was taken as a stability indicator and is plotted in Figure 3c.^[^ ^13^ ^]^ Typical degradation mechanisms observed during the catalyst AST include Ostwald ripening, nanoparticle agglomeration, morphological changes and metal dissolution. These degradation mechanisms can be mitigated when the electrocatalyst particle is positioned within the pores, as is the case for a portion of the nanoparticles of the KB300‐supported catalysts. As a result, the cell voltage drop remained minimal, with ≈40 mV during the 30k CVs of the AST of both KB300‐based seed‐mediated catalysts. Pt and Ir seed‐mediated Vulcan‐supported catalysts, along with the JM reference, exhibited a larger cell voltage drop between 70 and 80 mV along the test time. Closer inspection of the degradation rate revealed that the Ir seed‐mediated catalyst experienced an immediate significant voltage drop during the first 5000 CVs, followed by improved durability over the remainder of the AST. The non‐seed‐mediated sample displayed a very low initial cell voltage compared to all tested catalysts and revealed a very dramatic cell voltage drop during the AST of almost 100 mV. This difference in performance is attributed to the distinct structural and compositional characteristics of the non‐seed‐mediated catalyst as outlined above: Metal leaching and particle detachment are likely degradation causes, as Ostwald ripening is expected to be slower for the larger initial particles. These results further underscore the enhanced catalytic activity and durability of the seed‐mediated catalysts. After the AST, the more stable KB300‐based catalysts displayed the highest ECSAs with a retention of 70–75 % (Figure 3b). The JM reference and the two Vulcan‐based seed‐mediated catalysts showed a similar ECSA trend, retaining only slightly over 50% of their BOL ECSA. Representative CVs at BOL and EOL are presented in Figure S12 (Supporting Information) for all six tested catalysts. TEM images after MEA AST are shown in Figure S13 (Supporting Information). For all shown catalysts, the particle sizes grew as expected during the durability test. The particle size of the seed‐mediated samples increased from ≈4–5 to 6–7 nm. The non‐seed‐mediated NS‐L30‐C_V_ almost retained their particle size during the AST, only increasing slightly from 6.6 to 7.2 nm, all while the catalyst particles lost their octahedral shape.

We can conclude that KB300 support materials in combination with seed‐mediated alloy catalyst particles, appear to have a competitive advantage in terms of performance and durability over other combinations. The MA of all catalysts only decreased slightly after the AST. Both KB300‐based catalysts still displayed the highest MA, while the other four catalysts showed comparable MAs.

To understand the catalysts' dissolution in the electrochemical environment, *online* ICP‐MS measurements were conducted on the four catalysts with a Pt loading of 30 wt%. While trends from liquid electrochemical studies can provide insights, they must be interpreted with caution due to differences from MEA systems, such as temperature, local pH, ion mobility, proton conductivity, and other conditions.^[^ ^14^ ^]^ Before starting the electrochemical protocol, the metal dissolution induced by the first contact of the working electrode with the acidic electrolyte was measured, waiting till the ICP‐MS signal was stabilized. The electrochemical protocol, shown in Figure S14 (Supporting Information), consisted of the electrode surface conditioning (electrochemical activation) and 3 cyclic voltammograms at 5 mV s^−1^ to clearly resolve the dissolution events. Both steps mimic the RDE protocol. Thereafter, 4 cyclic voltammograms at 5 mV s^−1^ with a higher upper potential limit (0.95 V) were applied, followed by 3 trapezoidal‐like potential cycles with upper and lower potential limits of 0.60 and 0.95 V, respectively, holding each for 3 min and a slow transition between them at 5 mV s^−1^. This step was chosen to clearly resolve the dissolution events during the AST potential exclusion used in the MEA testing. Finally, a 1000 cycles AST (square wave potential cycles between 0.6 and 0.95 V with 3 s hold time) was applied to quantify the specific metal dissolution during the AST.

For all the catalysts under study, the most important Ni dissolution event takes place during the first contact between the working electrode and the acidic electrolyte, in line with our previous contribution.^[^ ^15^ ^]^ The origin of the intense Ni contact peaks shown in Figure S15 (Supporting Information) might be related to the chemical dissolution of the non‐pre‐leached catalysts surface, expecting a Pt‐rich surface.^[^ ^16^ ^]^ However, the data suggest that metal leaching during the first contact of the catalysts with the electrolyte is dependent not only on the support material but also the synthesis method (Figures S15 and S20, Supporting Information): PtS10‐L30‐C_KB_ presented the lowest amount of dissolved metals, significantly lower than for the other catalysts, underscoring the importance of the support material and nanoparticle placement in enhancing the catalyst stability. Additionally, the Ir seed‐mediated catalyst showed slightly higher specific dissolution of Pt and Ni compared to its Pt seed‐mediated counterpart, as well as over two times higher dissolution of Ir due to the higher Ir content. The non‐seed‐mediated sample showed higher levels of dissolved metals compared to PtS10‐L30‐C_V_ at the contact peak, indicating improved stability of the seed‐mediated samples.

During the electrochemical activation and in all cases, once the electrode potential is controlled and goes to the lower potential limit, sharp Pt dissolution peaks are visible in the specific dissolution profiles, see Figure S16 (Supporting Information), followed by Ir and Ni dissolution peaks. Such peaks decay through the potential cycling until the signals are stabilized. Interestingly, the specific dissolution profiles revealed that Pt is first leached out, and the Ir and Ni dissolution followed the Pt one. This observation might suggest that, after the possible early surface reconstruction during the contact peak, the partial or total dissolution of the Pt‐rich surface allows the underlying Ni and/or Ir sites, accessible to the acidic electrolyte, to be quickly oxidized and dissolved till the surface is stabilized. Such events induce structural and compositional transitions with a significant effect on the catalyst's performance.^[^ ^17^ ^]^

Following the electrochemical activation, slow triangular potentiodynamic cycles at 5 mV s^−1^ were performed to clearly resolve the transient metal dissolution behavior. We started using an upper potential limit of 0.925 V to mimic the RDE protocol (Figure S17, Supporting Information), and then the upper potential was increased to 0.95 V (used value for the AST, Figure S18, Supporting Information). In all cases, the trends are the same but with a slightly increased Pt dissolution using 0.95 V as the upper potential limit, confirming the well‐known upper potential limit effect.^[^ ^18^ ^]^ Looking at the specific Pt dissolution profiles in Figures S17 and S18 (Supporting Information), one can notice that the predominant Pt dissolution signal is centered at the cathodic scan. The origin of this cathodic peak is linked to the electrochemical reduction of the formed surface Pt oxides during the anodic scan (higher surface oxidation degree as the upper potential increases), which induces a surface and subsurface restructuring via the place exchange mechanism.^[^ ^18^ ^]^ In fact, PtS10‐L30‐C_KB_ presents broader Pt dissolution peaks, which can be attributed to its smaller mean particle size.^[^ ^19^ ^]^ In the case of the specific Ni dissolution profiles, an anodic and a predominant cathodic peak emerge, in agreement with the literature.^[^ ^20^ ^]^ The anodic peak might be related to the easy segregation of Ni during the surface oxidation, following the Ni dissolution proceeding after the cathodic Pt dissolution. For the specific Ir dissolution profiles, Figures S17 and S18 (Supporting Information) reveal two characteristic features for all samples, a cathodic and an anodic peak. While the cathodic dissolution event is related to the electrochemical reduction of IrO_x_ species (disruptive process of removal of oxygen from the crystal structure of surface oxides), the anodic dissolution event is linked to the formation of iridium oxide species, which might be amorphous (presence of low‐coordinated sites) and easily dissolved.^[^ ^21^ ^]^

**Figure 4:**
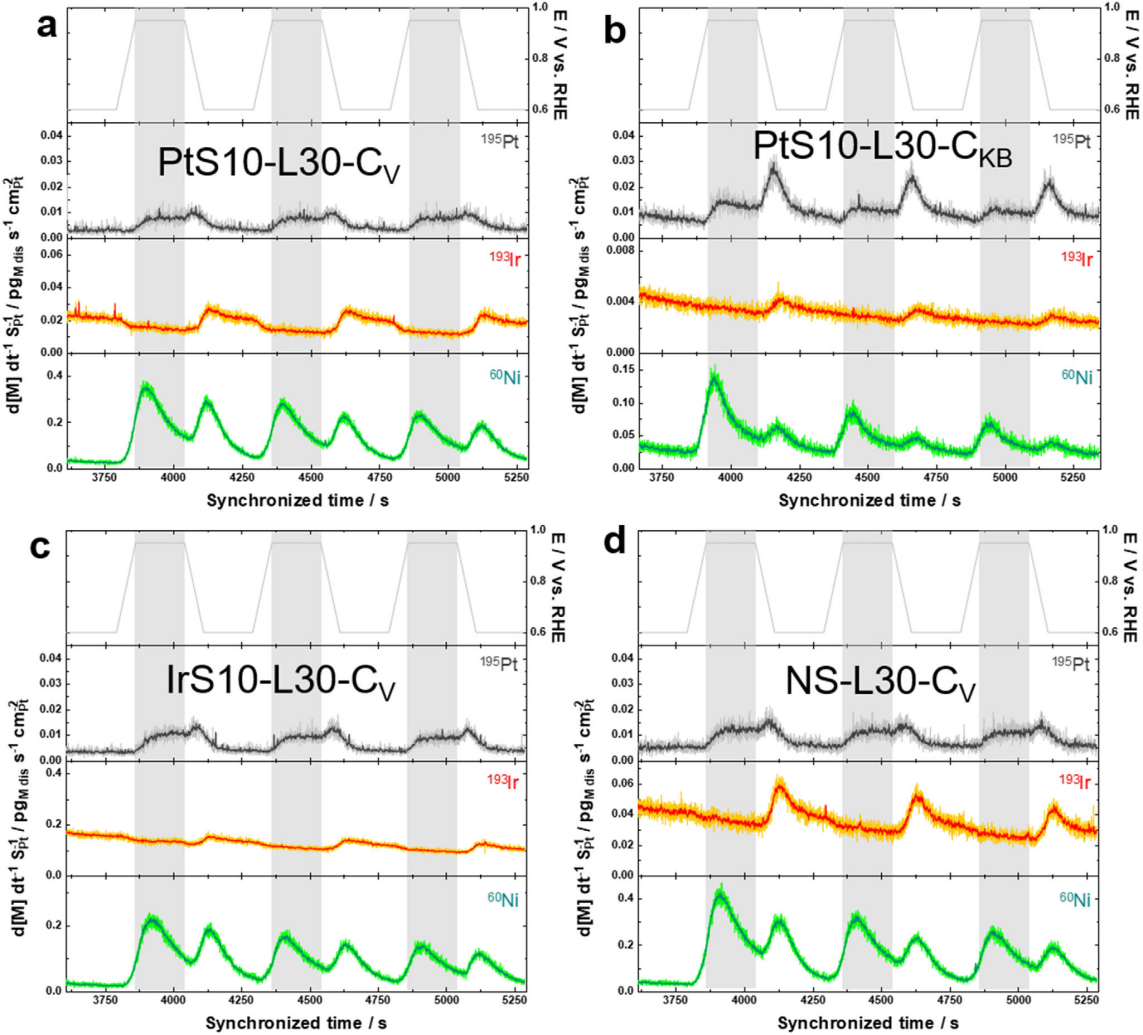
Online ICP‐MS signal of Pt (black, top), Ir (red, middle) and Ni (green, bottom) during trapezoidal wave CV protocol of a) PtS10‐L30‐C_V_, b) PtS10‐L30‐C_KB_, c) IrS10‐L30‐C_V_ and d) NS‐L30‐C_V_. The *y*‐axis ranges for the Ir and Ni signal change depending on the measured sample to better represent the metal dissolution response to the CV protocol.

Furthermore, the effect of the square potential wave used in the AST on the catalyst's dissolution was investigated. **Figure** **4** showcases the specific dissolution profiles of the series under study. The dissolution events that take place using this potential exclusion were clearly resolved and deconvoluted by holding the lower and upper potential limits (0.60 and 0.95 V, respectively) for 3 min, following a slow transition between them at 5 mV s^−1^. The specific Pt dissolution profiles shown in Figure 4 reveal an anodic dissolution signal as the electrode potential increases, which peaks as soon as the value of 0.95 V is reached, followed by a slow decay. This feature might be related to the formation and dissolution of amorphous surface oxides and/or metastable Pt–O_x_H_y_ species due to the slow Pt oxidation kinetics.^[^ ^22^ ^]^ Once a stable surface Pt oxide is formed, the dissolution signal decays due to the surface passivation.^[^ ^17c^ ^]^ Once the potential changes its direction to the lower potential limit, a predominant cathodic dissolution peak emerges, the origin of which is linked to the electrochemical reduction of the stabilized surface Pt oxides through the place‐exchange mechanism, i.e., the exchange of the original lattice sites of surface Pt atoms by O atoms. While it seems that Ir is only dissolved during the reduction scan, Ni is strongly dissolved during the anodic scan, followed by a subsequent cathodic dissolution, which follows the Pt one.

Regarding the metal dissolution during the AST, Figures S19 and S20 (Supporting Information) revealed that the PtS10‐L30‐C_KB_ catalyst showed the lowest dissolution of Ir and Ni, with the latter being an order of magnitude lower compared to the other catalysts. Furthermore, all four catalysts displayed similar dissolution of Pt during the AST. The other two seed‐mediated catalysts (PtS10‐L30‐C_V_ and IrS10‐L30‐C_V_) exhibited comparable Pt and Ni metal dissolution, while the Ir dissolution was higher for the Ir seed‐mediated catalyst. In contrast, the non‐seed‐mediated catalyst (NS‐L30‐C_V_) demonstrated the highest Ir dissolution despite significantly lower amounts of Ir in the catalyst compared to the Ir seed‐mediated catalyst. This disparity in Ir dissolution could potentially account for the lower durability of the non‐seed‐mediated catalysts, as Ir is not retained in the catalyst hindering the stability of the nanoparticles.

The *online* ICP‐MS measurements also elucidated that the superior durability of PtS10‐L30‐C_KB_ during PEMFC measurements can be correlated to the enhanced stability of the catalyst nanoparticles with lower dissolution of Ir and Ni. Overall, it may be concluded from the results from *online* ICP‐MS measurement that the seed‐mediated synthesis route offers higher stability, as evidenced by substantially lower metal dissolution compared to the non‐seed‐mediated catalyst.

## 3 Conclusion

In this study, a series of seed‐mediated nanoparticles using platinum and iridium seeds were synthesized, and their structure‐activity‐stability relations were analyzed with respect to the catalytic ORR process in both idealized liquid electrolyte and realistic PEMFC environments. A range of ex‐situ and in‐situ analytical techniques aided in gaining insights into their pre‐catalytic composition, morphology, surface properties, as well as into their surface and bulk characteristics during catalytic testing. Electrochemical performance and stability were assessed in three distinct environments, including liquid RDE measurements, online ICP‐MS dissolution analysis, and single PEMFC MEA testing. The results showed that the seed‐mediated samples exhibited enhanced performance and durability under fuel cell operation. The performance and durability were further increased for the seed‐mediated samples prepared on porous carbons. A comparative analysis between Pt and Ir seed‐mediated catalysts revealed that Pt seeds resulted in enhanced catalytic performance compared to Ir seeds in MEAs. Online ICP‐MS analysis showed that the metal dissolution behavior is strongly influenced by the synthesis method and carbon support type. The use of KB support in the seed‐mediated synthesis route significantly improved catalyst stability by reducing Ir and Ni dissolution.

The results suggest that sophisticated catalysts, as from our seed‐mediated synthesis route, offer further performance and stability enhancements under fuel cell operating conditions. This also holds true for higher loaded catalysts (≥40 wt%) while maintaining structural advantages. Further performance improvements could be achieved by utilizing seed‐mediated catalysts supported on novel carbon materials, which integrate the beneficial properties of both porous and solid carbon. Such a hybrid or advanced carbon support would harness the high surface area and stability of porous carbon, while also providing the advantage of solid carbons under high current densities.

## Conflict of Interest

The authors declare no conflict of interest.

## Author Contributions

L.P. and T.M. contributed equally to this work. L.P. and T.M. analyzed the data and composed the manuscript. L.P. synthesized the catalysts and performed the RDE measurements. T.M. conducted MEA and MEA AST measurements. C.A.C.R. performed online ICP‐MS tests. A.G. and J.L. assisted in the catalyst synthesis. J.S. carried out the XPS measurements. M.K., X.W., S.M., and S.S. performed the TEM characterization. M.H. carried out the STEM measurements. D.J. and P.S. supervised the work. All authors participated in the discussion.

## Supporting information



Supporting Information

## Data Availability

The data that support the findings of this study are available from the corresponding author upon reasonable request.
