# Supplementary material for: From Seeds to Cell: Improving PEMFC Performance and Durability by Seed‐Mediation Synthesis for PtNiIr ORR Nanocatalysts
Source: Adv Sci (Weinh). 2025 May 20;12(29):e05958. doi: 10.1002/advs.202505958 (PMC12362820; doi:10.1002/advs.202505958)
Supplement: Supplementary file 1 — Supporting Information [file ADVS-12-e05958-s001.docx]

**Supporting Information**

**From Seeds to Cell: Improving PEMFC Performance and Durability by Seed-Mediation Synthesis for PtNiIr ORR Nanocatalysts**

**Lujin Pan*^,^** **^a^, Thomas Merzdorf*^,^** **^a^, Carlos A. Campos-Roldàn^b^, An Guo ^a^, Jiasheng Lu ^a^, Johannes Schmidt ^a^, Marc Heggen^c^, Malte Klingenhof ^a^, Xingli Wang ^a^, Sebastian Möhle^a^, Sören Selve^d^,** **Deborah Jones^b^, Peter Strasser^+, a^**

^a^ Department of Chemistry, Technische Universität Berlin, 10623 Berlin, Germany

^b^ ICGM, Univ. Montpellier, CNRS, ENSCM, 34095 Montpellier, France

^c^ Ernst Ruska-Centre for Microscopy and Spectroscopy with Electrons, Forschungszentrum Juelich GmbH, 52425 Juelich, Germany

^d^ Center for Electron Microscopy (ZELMI), Technische Universität Berlin, 10623 Berlin, Germany

* The authors contributed equally.

+ Corresponding author.

**Experimental part**

**Synthesis**

Pt seed and Ir seed synthesis

58.0 mg of platinic chloride (IV) hexahydrate [H_2_PtCl_6_∙6H_2_O] (99.95%, Thermo Scientific) was weighed into a 50 mL centrifuge tube, and 1 mL of Milli-Q water was added to dissolve the platinum precursor. Meanwhile, 200 mg of carbon powder was mixed with 3 mL isopropanol and 2 mL Milli-Q water. This suspension was then treated with a horn sonicator to ensure thorough distribution and mixing with the precursors. The precursor solution was added dropwise to the carbon suspension. After the addition, the mixture was sonicated in an ice bath for 15 minutes to achieve better distribution. Immediately after sonication, the tube containing the carbon and platinum precursor was cooled with liquid nitrogen bath and frozen for 30 minutes. The frozen mixture was then transferred to a freeze dryer and dried overnight. After the drying process, the resulting powder was granulated using an agate mortar and then transferred into a furnace tube. This tube was mounted in a vertical furnace with a shaker, which enabled dispersion during heating. The reaction took place in a 4% H_2_/Argon atmosphere at 200 °C for 2 hours. After the reduction, the powder was used as carbon with Pt seed for further synthesis.

For Ir seed, 59.3 mg IrCl_6_∙xH_2_O (99.9 %, Sigma Aldrich) was used as precursor to produce Ir seed supported on carbon using the same procedure as for the Pt seeds.

PtNiIr synthesis

288.0 mg (732.0 μmol) of Pt(acac)_2_ (Pt 48.0% min., Alfa Aesar), 144.0 mg (560.4 μmol) of Ni(acac)_2_ (95%, Sigma Aldrich) and 2160.0 mg (17.7 mmol) of benzoic acid (99.5%, Sigma Aldrich) were put into an autoclave glass liner containing a magnetic stir bar. Separately, a suspension of 80 mg of carbon/ Pt seed or carbon/ Ir seed in 40 mL of DMF was prepared by ultrasonication using a Branson Sonifier® SLPe for 15 minutes. This carbon suspension was then added to the autoclave glass liner, and the mixture was placed in an ice bath and ultrasonicated for 30 minutes.

The autoclave, a 100 mL/100 bar Model I Roth, was sealed with a PTFE ring and heated to 160 °C. The reaction mixture was stirred and maintained at this temperature for 12 hours. After the reaction, the autoclave was cooled to room temperature and opened. Subsequently, 12.0 mg (30.5 μmol) of Pt(acac)_2_, 6.0 mg (23.3 μmol) of Ni(acac)_2_, and 8.8 mg (18.0 μmol) of Ir(acac)_3_ (97%, Sigma Aldrich) were dissolved in 5 mL of DMF by sonication for 5 minutes.

The dissolved precursor solution was transferred into the glass liner and ultrasonicated in a sonication bath for 40 minutes. The mixture was then transferred back into the autoclave, which was heated to 180 °C for 48 hours. After cooling to room temperature, the autoclave was opened, and the product was washed and dried overnight in a vacuum freeze dryer.

**ICP-OES**

An inductively coupled plasma optical emission spectroscopy (ICP-OES) analysis was conducted using a 715-ES-ICP analysis system (Varian) to determine the elemental composition of the synthesized particles. The selected wavelengths for concentration determination were 203.604 nm, 212.863 nm, 214.424 nm, 217.468 nm, 265.945 nm, and 306.471 nm for Pt; 216.555 nm, 221.648 nm, 222.486 nm, 227.021 nm, 230.078 nm, and 230.299 nm for Ni; and 204.419 nm, 205.116 nm, 212.618 nm, and 224.268 nm for Ir.

**XPS**

X-ray photoelectron spectroscopy (XPS) measurements were performed using a ThermoScientific K‑Alpha^+^ X-ray Photoelectron Spectrometer. All samples were analysed using a microfocused, monochromated Al K α X-ray source (1486.68 eV; 400 μm spot size) while the analyser had a pass energy of 50 eV. To prevent any localized charge buildup during analysis the K-Alpha^+^ charge compensation system was employed at all measurements. The peak fitting was performed using Avantage software.

**TEM**

Transmission electron microscopy (TEM) images were acquired using a FEI Tecnai G2 20 S-TWIN microscope equipped with a LaB_6_ cathode, operating at an acceleration voltage of 200 kV and a resolution limit of 0.24 nm. Samples were dispersed in isopropanol and drop-cast onto a 400-mesh copper (Cu) grid.

**STEM**

Scanning transmission electron microscopy (STEM) studies were conducted using a Hitachi High-Technologies HF5000 environmental STEM, equipped with secondary electron, dark field, and bright field detectors. The microscope was operated at 200 kV and is equipped with a Cs-probe corrector.

**RDE**

Ink preparation

To prepare the ink, 1.7 mg of catalyst with 30 wt% Pt or 1.2 mg of catalyst with 40 wt% Pt was added to 1.99 mL of ultrapure water, 5 µL of Nafion (5 wt%), and 0.5 mL of isopropanol (≥ 99.5%). The mixture was then subjected to horn ultrasonication for 30 minutes. Subsequently, 10 µL of the ink was placed on a glassy carbon (GC) rotating disk electrode (diameter Ø = 5 mm), resulting in a Pt loading of around 10 µg cm⁻². The film was then dried at 50 °C for 10 minutes.

Electrochemical characterization

For the electrochemical characterization, a conventional three-electrode cell was used, consisting of a reference electrode (MMS Hg/Hg₂SO₄, calibrated potential E = -0.725 V_RHE_), a glassy carbon working electrode and a Pt mesh counter electrode (Pt furled mesh, 5x5 cm²). The working electrode was always immersed in the electrolyte under potential control at 0.05 V_RHE_. Electrochemical measurements were conducted in 0.1 M HClO₄ (99.999% trace metal basis, Sigma Aldrich). All measurements were performed using BioLogic Science Instruments potentiostats SP-150 and SP-200. The gases used had a purity of 99.998% for oxygen, 99.999% for nitrogen, and 99.999% for hydrogen. Electrochemical activation was performed by cyclic voltammetry (CV) between 0.05 and 0.925 V_RHE_ at a scan rate of 100 mV s⁻¹ for 50 cycles under a nitrogen atmosphere. Subsequently, three additional cycles were applied within the same potential range at a scan rate of 20 mV s⁻¹. The third cycle was used to determine the electrochemical catalytic surface area (ECSA) from hydrogen underpotential deposition (H_upd_) via charge integration of the hydrogen ad-desorption region Q_H_. The measured Q_H_ value was normalized with respect to the theoretical value of Q_H_ ^theo^ = 210 µC cm⁻².The catalytic activity of the catalysts was assessed using linear sweep voltammetry (LSV). The measurement was performed between 0.05 and 1.0 V_RHE_, using a scan rate of 20 mV s⁻¹ for three cycles and a scan rate of 5 mV s⁻¹ for one cycle, all at a rotation speed of 1600 rpm.

The kinetic currents were calculated using the Koutecký-Levich equation (eq. 1):

$1/j$ = $1/jk$ + $1/jd$ [1]

where j was measured at 0.9 V_RHE_, j_d_ was determined in the diffusion-limited current region, and j_k_ is the calculated kinetic current density. Both currents obtained from the test were IR-corrected, where the resistance R was determined by potential electrochemical impedance spectroscopy at 0.5 V_RHE_, and then the background current, measured from the cathodic voltammetry sweep in N₂-saturated electrolyte at 0.4 V_RHE_, was subtracted.

The mass activity was calculated based on the kinetic current normalized by Pt weight loading, and the specific activity was normalized by the H_upd_-ECSA.

Stability test

Following the electrochemical activation, impedance spectrometry, and activity measurements, a stability test was carried out. This involved potential cycling between 0.6 and 0.925 V_RHE_ at a scan rate of 100 mV s^-1^ in an N_2_ saturated electrolyte for 10,000 cycles. After the AST the ECSA and MA was measured and calculated again.

CO stripping

CO stripping experiments were conducted subsequent to the measurements. Either directly after the electrochemical characterization or for samples which were subjected to an AST after the stability protocol. This sequencing is necessitated by the potential alteration in surface composition resulting from the interaction between surface Pt or Ni atoms and CO during CO stripping. Consequently, CO stripping serves as the final electrochemical test step in the RDE procedure.

During CO stripping, a potential of 0.05 V_RHE_ was applied at 400 rpm in N2 saturated electrolyte. Subsequently, CO was introduced into the electrolyte to cover the electrode surface with CO. Afterwards excess CO was removed from the electrolyte by saturating again with N2 gas. Following this, three CVs were recorded at 20 mV s^-1^ between 0.05 V_RHE_ and 1 V_RHE_ without rotation.

During the first scanning CV, the oxidation of surface adsorbed CO (assumed as one monolayer) took place, with the corresponding oxidation represented by the CO stripping peak. The IR-corrected current of the first positive scan was then subtracted by that of the second positive scan, which served as the background current. Subsequently, the integral of the peak was calculated and translated based on the assumption of one layer of CO occupying a charge of 420 µC cm^-2^.

**MEA preparation^1^**

For the cathode catalysts, an ink consisting of 0.5 wt% solid fraction with an I/C-ratio of 0.6 in a water isopropanol mixture (10:90 wt%) was homogenized. Nafion (20 wt%) was used as ionomer. The resulting suspension was sprayed onto the microporous side of a gas diffusion layer (GDL; 22BB, Sigracet) using our spraycoater (Exactacoat, Sono-Tek) to obtain a catalyst loading of 0.15 mg_Pt_/cm². For the anode, a commercial 50 wt% Pt on Carbon catalyst from Umicore (Elyst Pt50) was used with an I/C-ratio of 0.6 in a water-isopropanol mixture (10:90 wt%) to produce an ink with a solid fraction of 1 wt%. Nafion (20 wt%) was used as ionomer. The ink was sprayed onto the microporous side of the GDL (22BB) with a loading of 0.1 mg_Pt_/cm². An extra coating of Nafion was sprayed onto each GDL with a 1 wt% Nafion solution. A 10 cm² anode and a 10 cm² cathode were placed on each side of the Nafion membrane (NR-212) together with gaskets and hot-pressed at 146 °C for 4 min at 1.7 MPa_abs_. The MEA was then mounted into the 10 cm² cell (Fuel Cell Technologies), tightened with 10 Nm torque and connected to our fuel cell test stand (Fuel Cell Technologies).

**MEA break-in and AST protocols^1^**

The break-in procedure consists of 10 loops at different potentials, 0.6 V, OCP and 0.85 V for 45 min, 5 min and 10 min respectively at 80 °C and 100% RH in H_2_/air atmosphere. Afterwards, different characterizations are measured at 30% RH (85 °C) and 100% RH (80 °C), impedance spectroscopy to determine the high frequency resistance and the proton resistance, cyclic voltammograms for the cross-over current and the ECSA_H-upd_ and oxygen polarization curves for the mass activity. For the accelerated stress test (AST), an adjusted protocol for the catalyst degradation from the US Department of Energy was used.^2^ This AST consists of 30,000 square wave voltammograms between 0.6 and 0.95 V, with a hold time of 3 s in H_2_/N_2_ atmosphere with minimal flows of 50 ml/min and 800 ml/min at the anode and cathode, respectively. After 0, 1,000, 5,000, 10,000 and 30,000 CVs, H_2_/air polarization curves were conducted at 80 °C and 100% RH with 150 kPa backpressure and stoichiometries of 1.5 and 2 at the anode and cathode, respectively, with the same minimal flows. The polarization curves were corrected for the cross-over current and the HFR of the cell and setup without catalyst layers.

**Electrochemical online ICP-MS**

A commercially available electrochemical flow cell (BASi, MF-1092, cross-flow cell) was used, which consist of two glassy carbon disks (3 mm) aligned in series embedded into PEEK material. The first disk was used as counter electrode, and the working electrode is in the direction of the electrolyte flow. An Ag/AgCl reference electrode was used, which was calibrated relative to the reversible hydrogen electrode (RHE) before any measurement. The electrocatalytic ink formulation was the same as used for the RDE measurements, but *ca*. 3.45 µL of the ink were carefully deposited onto the glassy carbon disk. The electrolyte (N_2_-saturated 0.1 M HClO_4_) was pumped through the flow cell using a peristaltic pump Watson Marlow 120S at *ca.* 400 μL min^-1^. Measurements were performed at 25±2 °C.

The electrochemical flow cell was connected to an Agilent 7900 ICP-MS equipped with a Micromist/Scott nebulizer. The ^195^Pt, ^193^Ir and ^60^Ni ions were detected with 0.1 s integration time per point. Calibration curves were done before each measurement using daily prepared standard solutions of ^195^Pt, ^193^Ir and ^60^Ni (SCP Science, Canada).

Prior to each measurement, Mili-Q water was pumped to the flow cell during 5 minutes. After that, the electrolyte was pumped to the flow cell, acquiring the metal dissolution during the first contact with the acidic electrolyte. Next, the ICP signal and the open circuit potential (OCP) were stabilized. The electrochemical protocol, shown in Figure S8, consisted in the surface conditioning or electrochemical activation by cycling the electrode potential between 0.05 and 0.925 V_RHE_ at a scan rate of 100 mV s⁻¹ for 20 cycles. Then, three cycles under the same potential window were applied at a scan rate of 5 mV s⁻¹ to resolved the transient metal dissolution mimicking the RDE conditions. Afterwards, three additional cycles at 5 mV s⁻¹ were applied increasing the upper potential limit to 0.95 V_RHE_, holding the electrode potential to 0.6 V_RHE_ at the end of this step. Next, three trapezoidal-like potential waves were applied (inspired from the US Department of Energy trapezoidal-wave protocol).^2^ This step consists of potential cycling between 0.60 - 0.95 V_RHE_, both potential limits were held for 3 minutes and switched at 5 mV s⁻¹. Finally, a 1000 potential cycles AST was applied using trapezoidal-like potential cycles between 0.60 - 0.95 V_RHE_. Both potential limits were held for 3 seconds and switched at 0.7 V s^-1^.


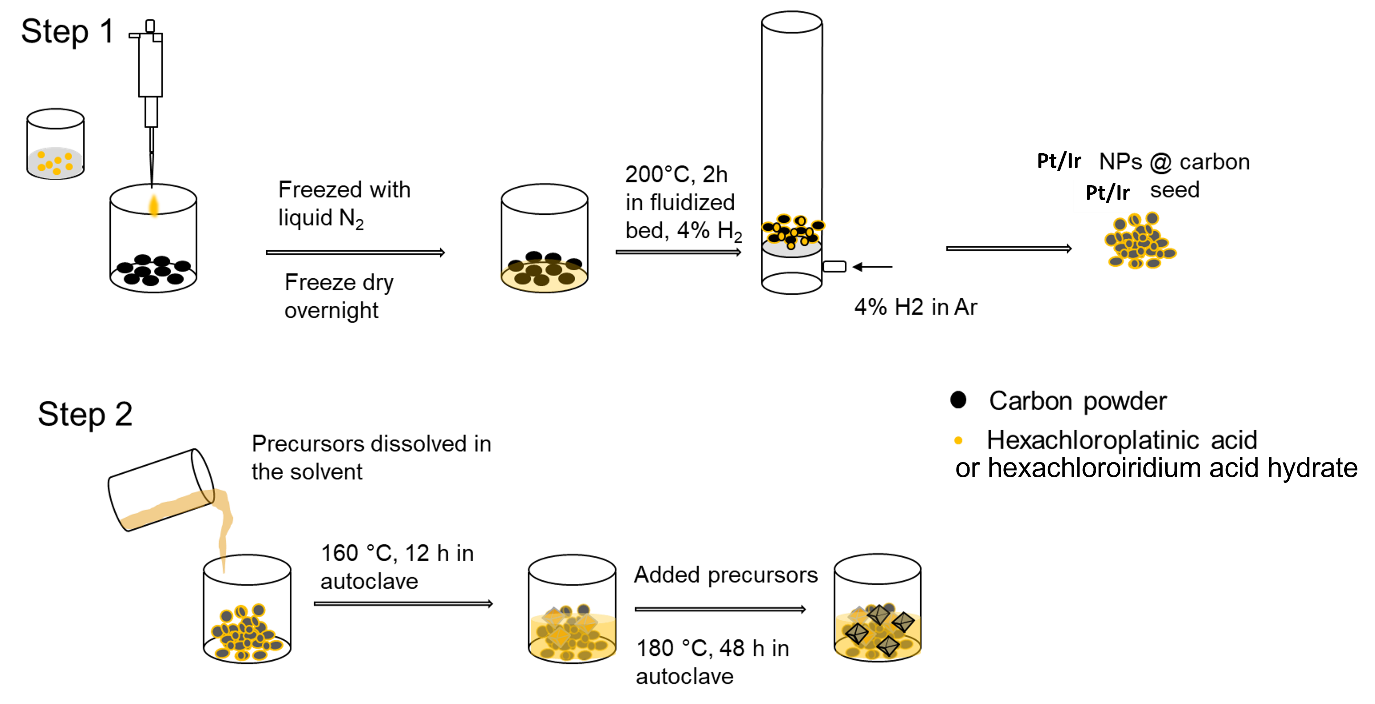


Figure S1: Seed-mediated synthesis procedure.


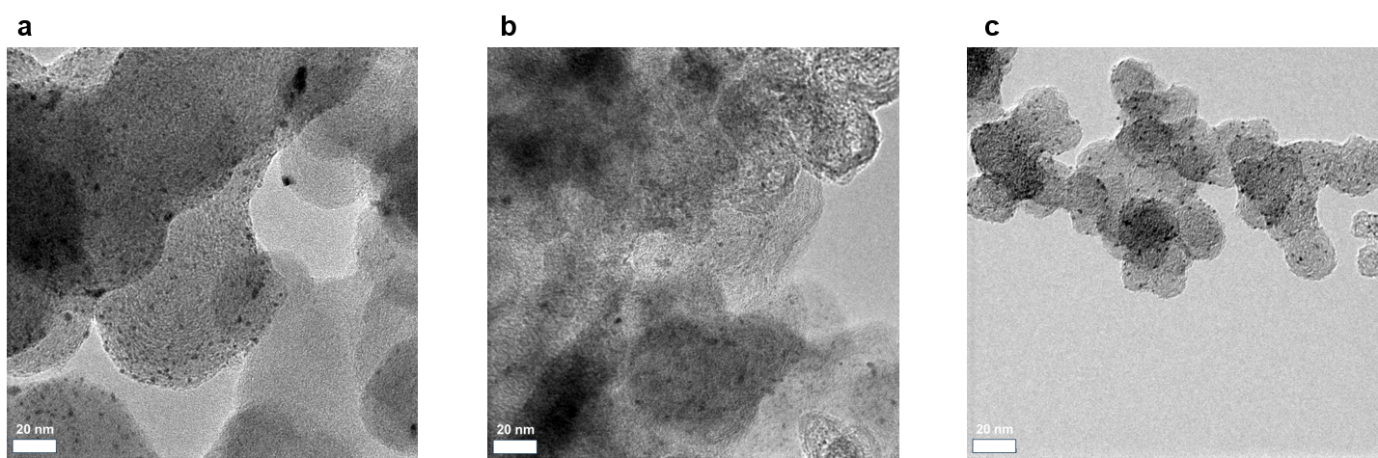
Figure S2: TEM images of a) Pt seed on carbon Vulcan, b) Pt seed on carbon Ketjen Black, c) Ir seed on carbon Vulcan. Scale bar is 20 nm.

Table S1: Composition of samples from ICP-OES data.


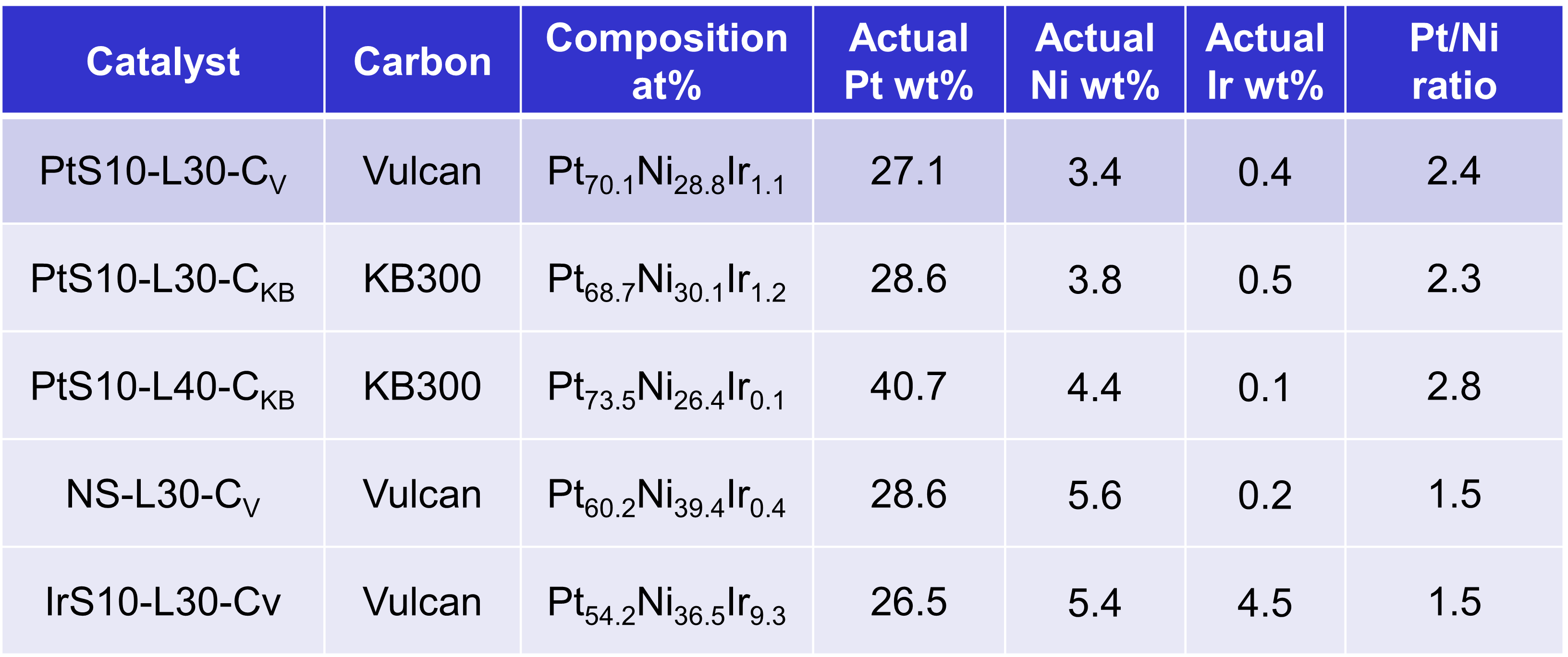


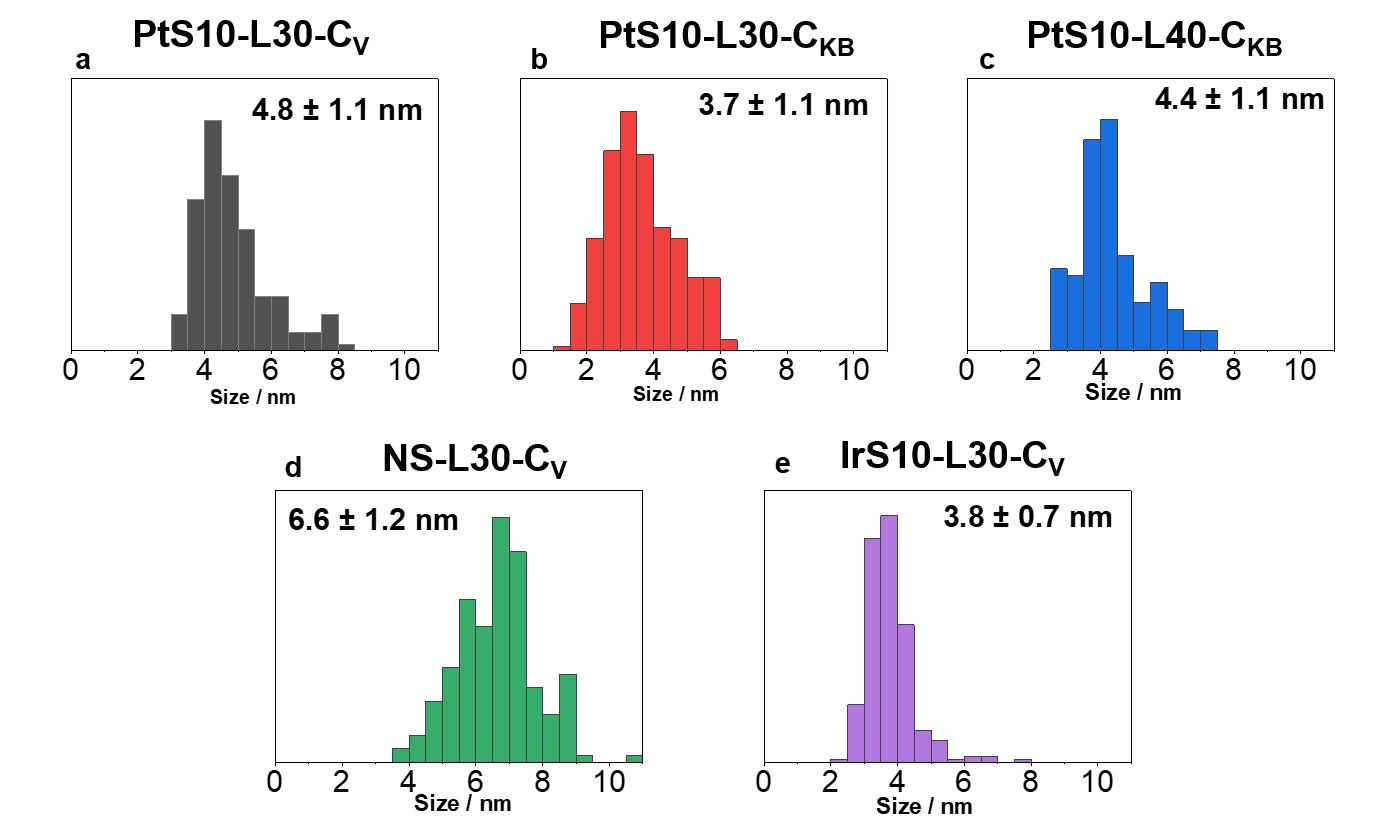


Figure S3: histogram of the particle size distribution from TEM images.


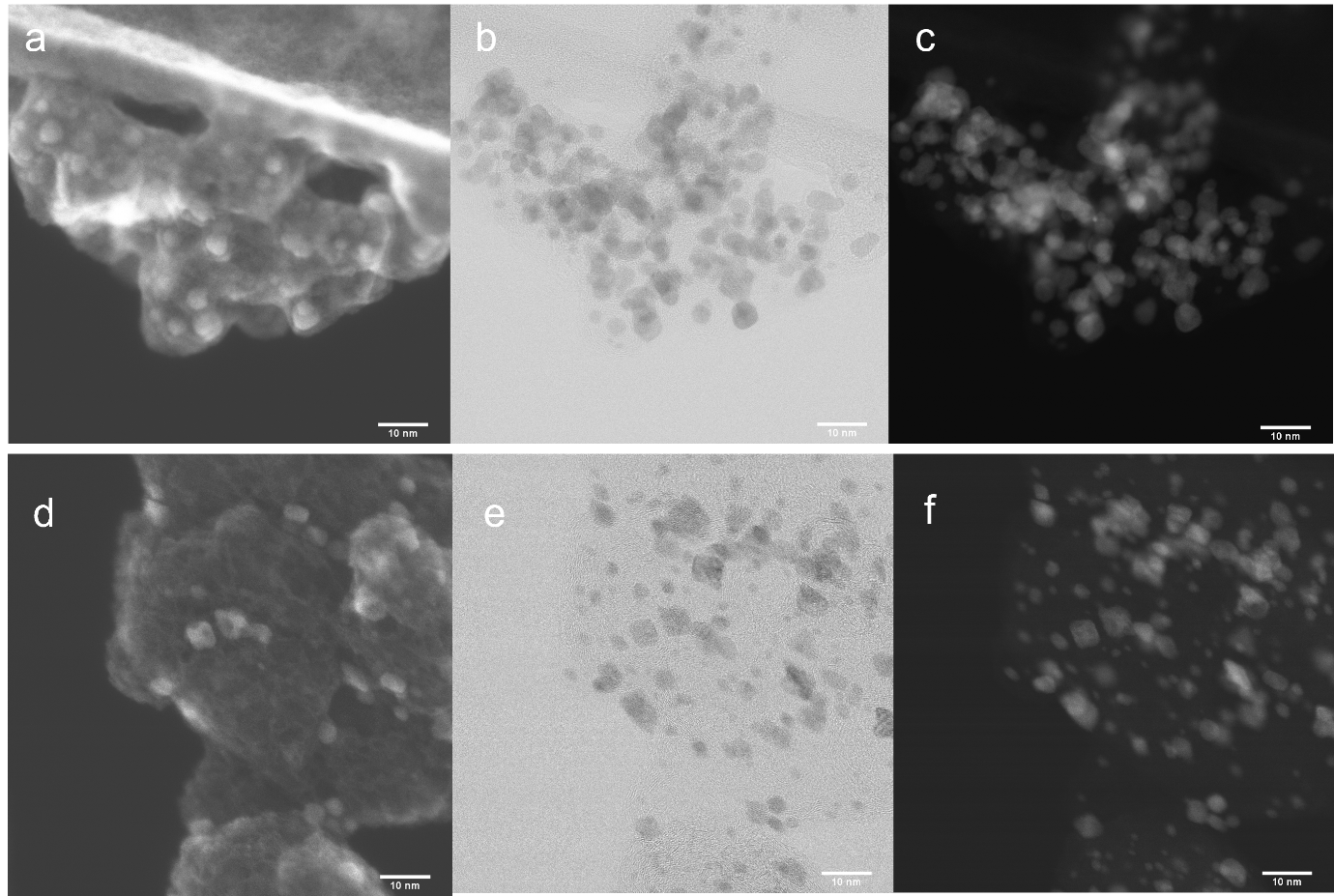


Figure S4: Correlative scanning transmission electron microscopy (STEM) images of a-c) PtS10-L30-C_V_, and d-f) PtS10-L30-C_KB_. a,d) Secondary electron (SE), b,e) bright field (BF), and dark field (DF) images.


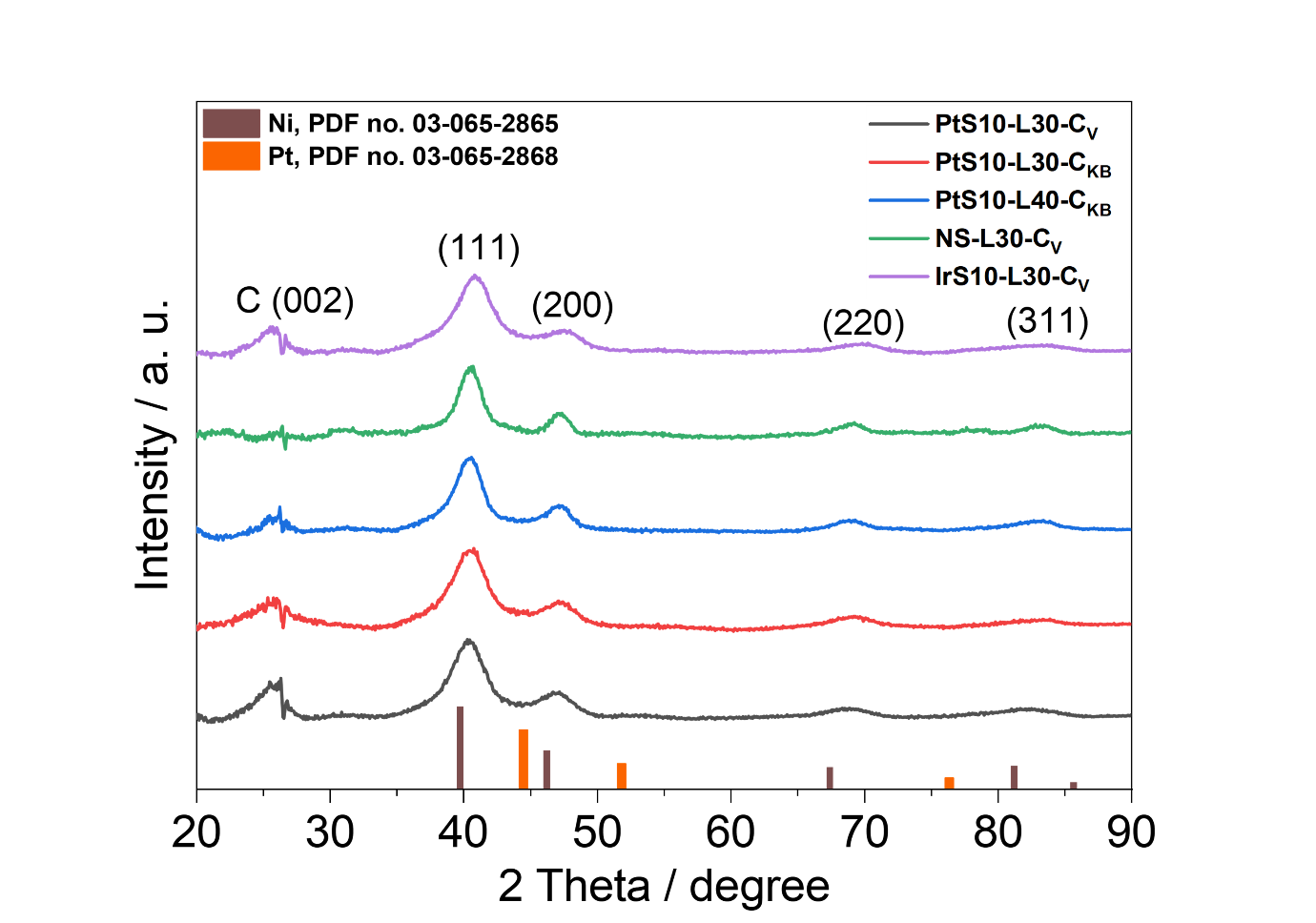


Figure S5: XRD patterns of as prepared PtS10-L30-C_V_ (black), PtS10-L30-C_KB_ (red), PtS10-L40-CK_B_ (blue), NS-L30-C_V_ (green) and IrS10-L30-C_V_ (violet).


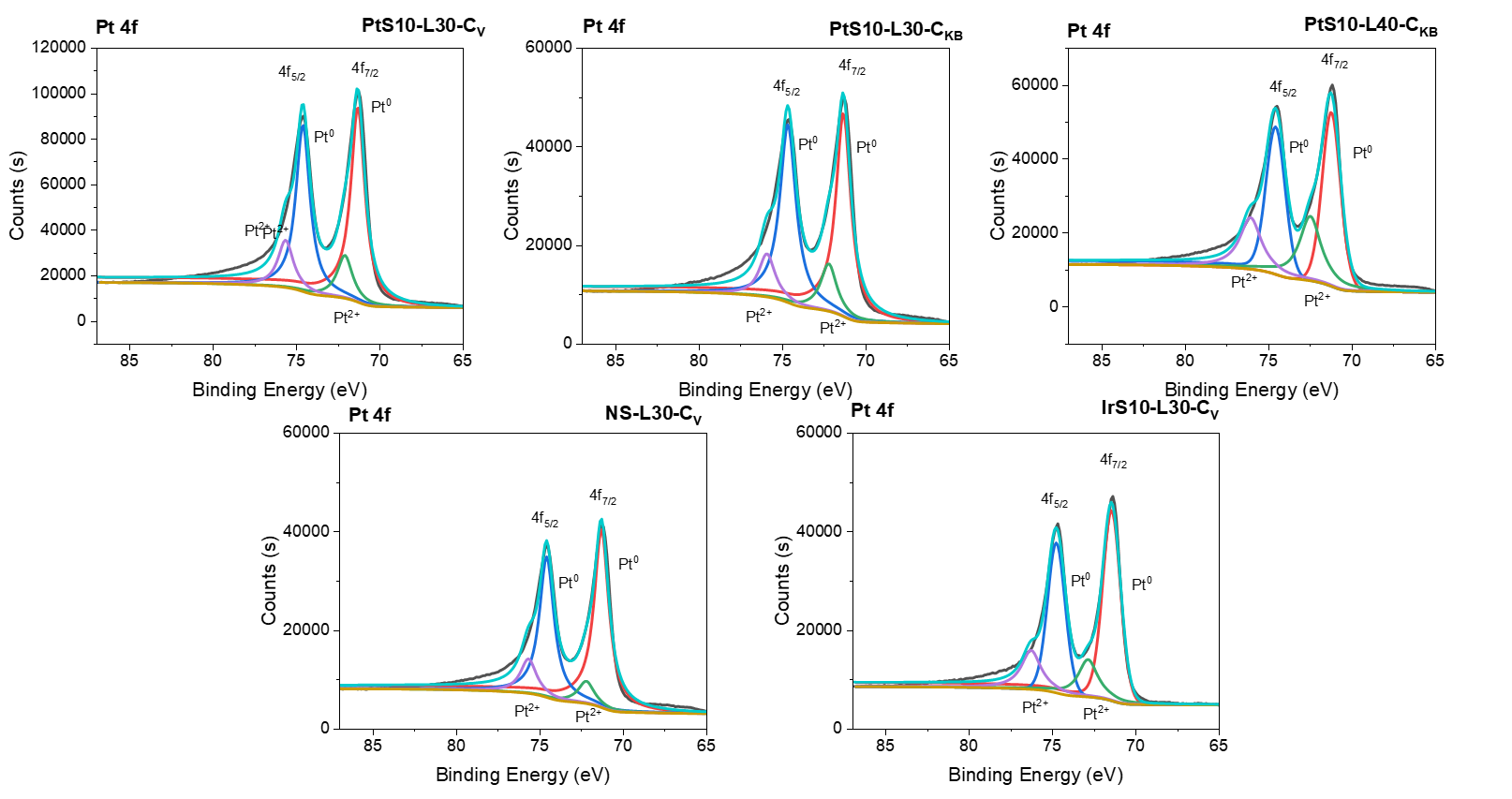


Figure S6: Pt 4f XPS fitting data.


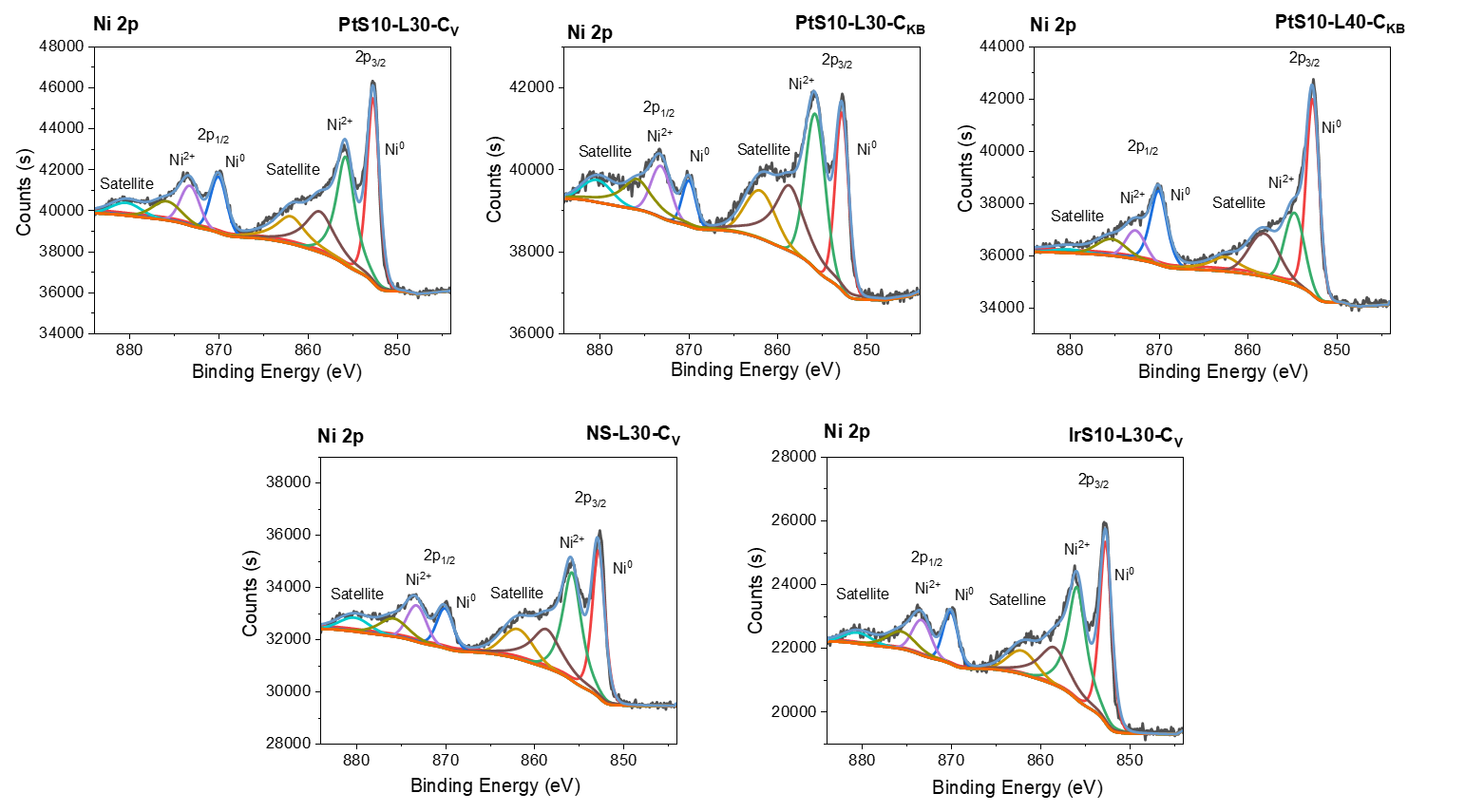


Figure S7: Ni 2p XPS fitting data.


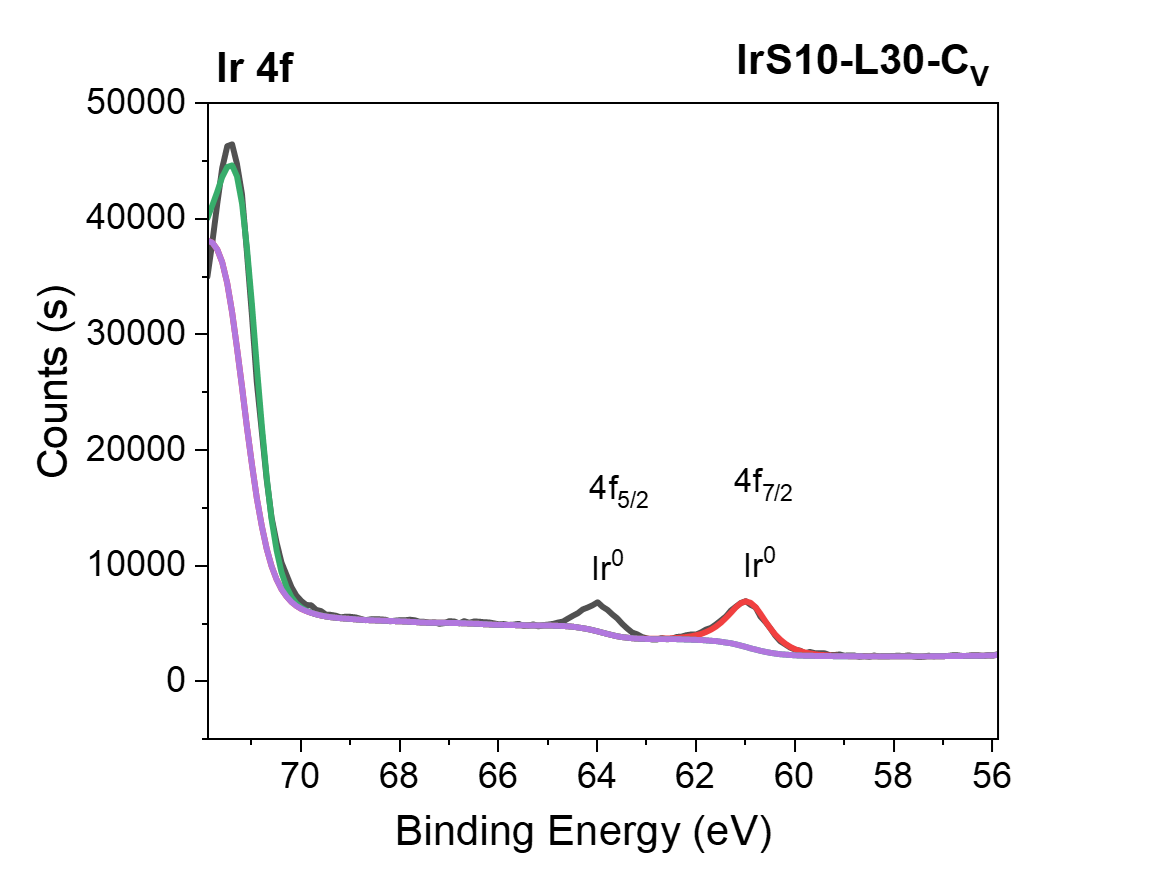


Figure S8: Ir 4f XPS fitting data of IrS10-L30-C_V_. The peak at ~73 eV corresponds to Pt 4f (from Pt in the sample); other peaks are assigned to Ir 4f_7/2_ and Ir 4f_5/2_.


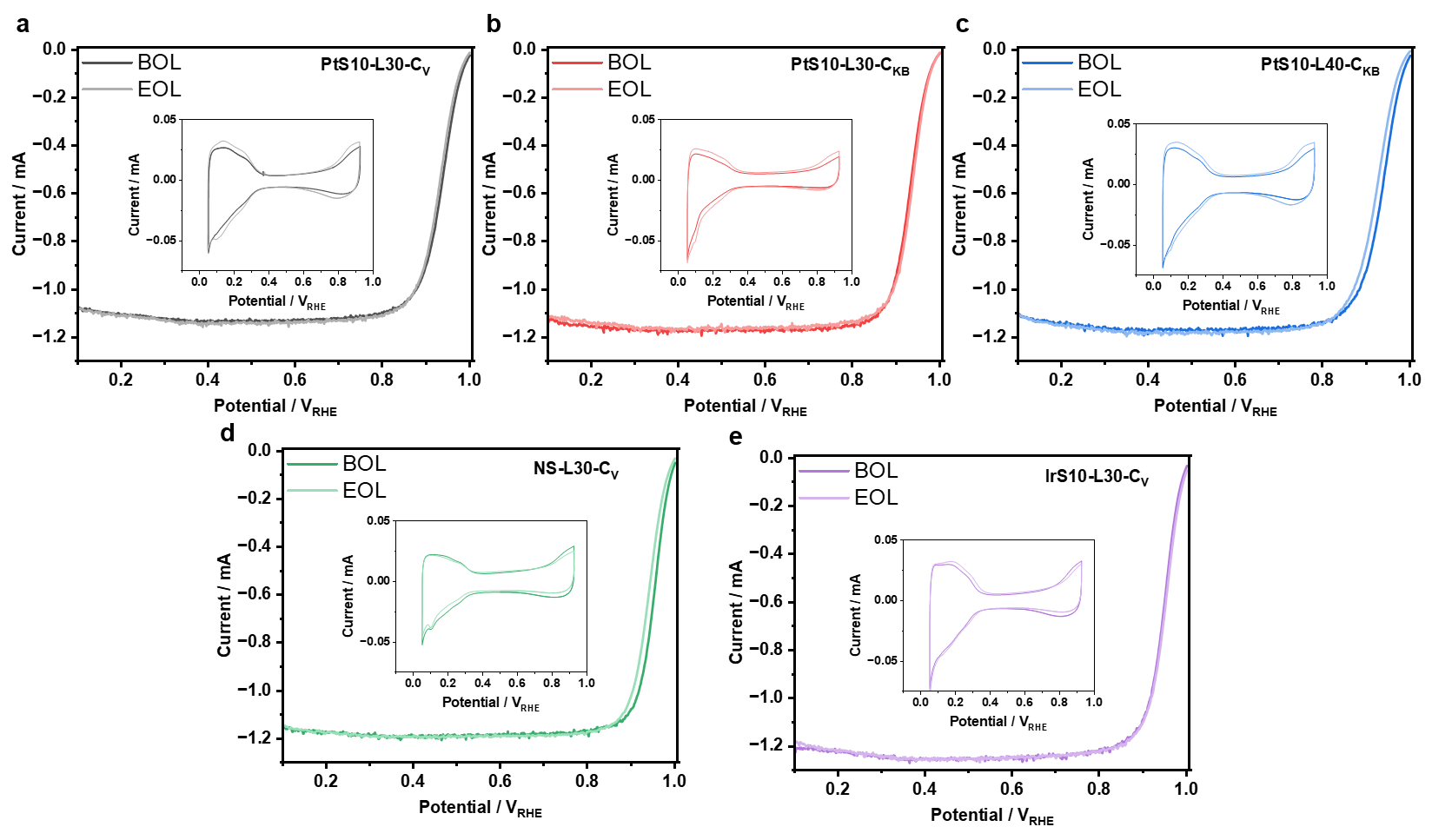


Figure S9: RDE CV and LSV curves recorded before (BOL) and after (EOL) AST for the investigated catalysts.


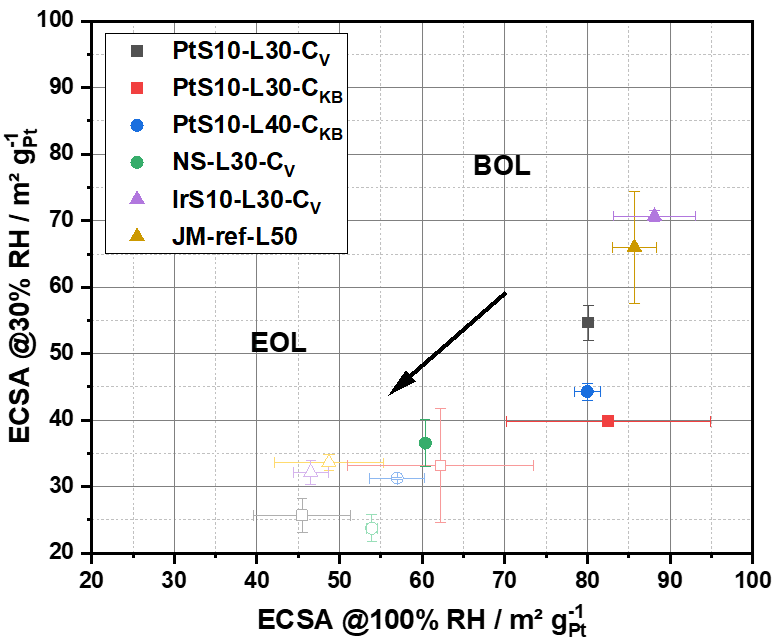


Figure S10: Electrochemical characterization of all catalysts in MEA. ECSA_H-upd_ at low (30%) and high (100%) RH before (darker and filled symbols) and after (lighter and hollow symbols) AST.


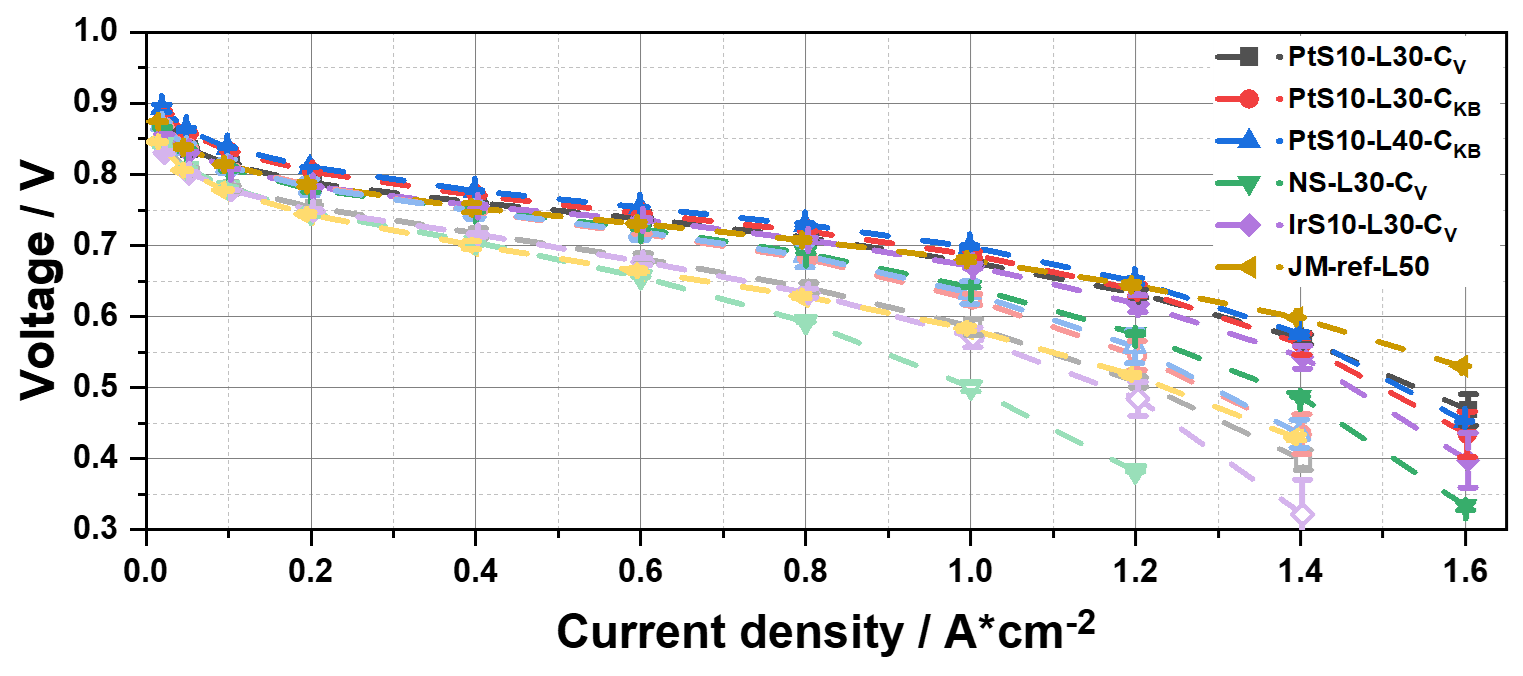


Figure S11: Comparison of polarization curves before (BOL, filled symbols) and after (EOL, hollow symbols and lighter colors) AST of S10-L30-C_V_ (black), S10-L30-C_KB_ (red), S10-L40-C_KB_ (blue), NS-L30-C_V_ (green), IrS10-L30-C_V_ (violet), JM 50 wt%Pt (gold).


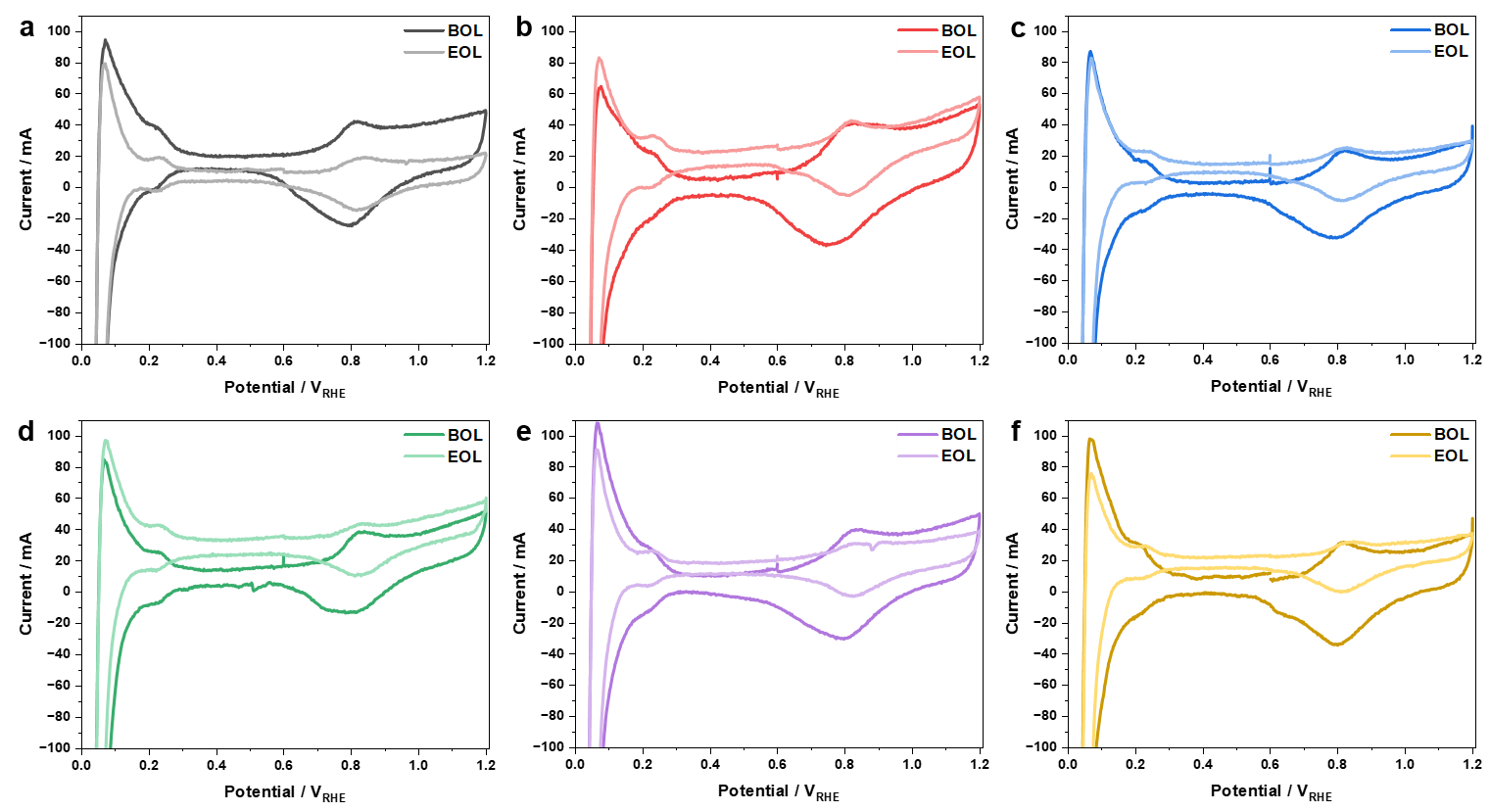


Figure S12: Comparison of CVs before (BOL) and after (EOL) AST of: a) S10-L30-C_V_ (black); b) S10-L30-C_KB_ (red); c) S10-L40-C_KB_ (blue); d) NS-L30-C_V_ (green); e) IrS10-L30-C_V_ (violet); f) JM 50 wt%Pt (gold).


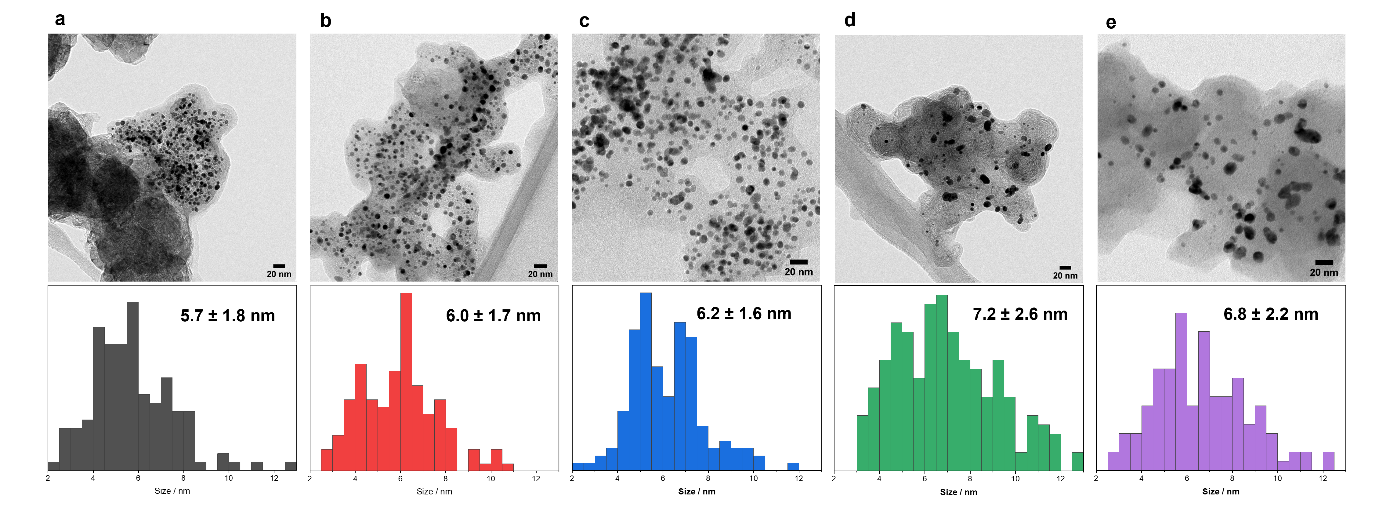


Figure S13: TEM images (top) and particle size distribution (bottom) after MEA AST of: a) S10-L30-C_V_ (black); b) S10-L30-C_KB_ (red); c) S10-L40-C_KB_ (blue); d) NS-L30-C_V_ (green); e) IrS10-L30-C_V_ (violet).


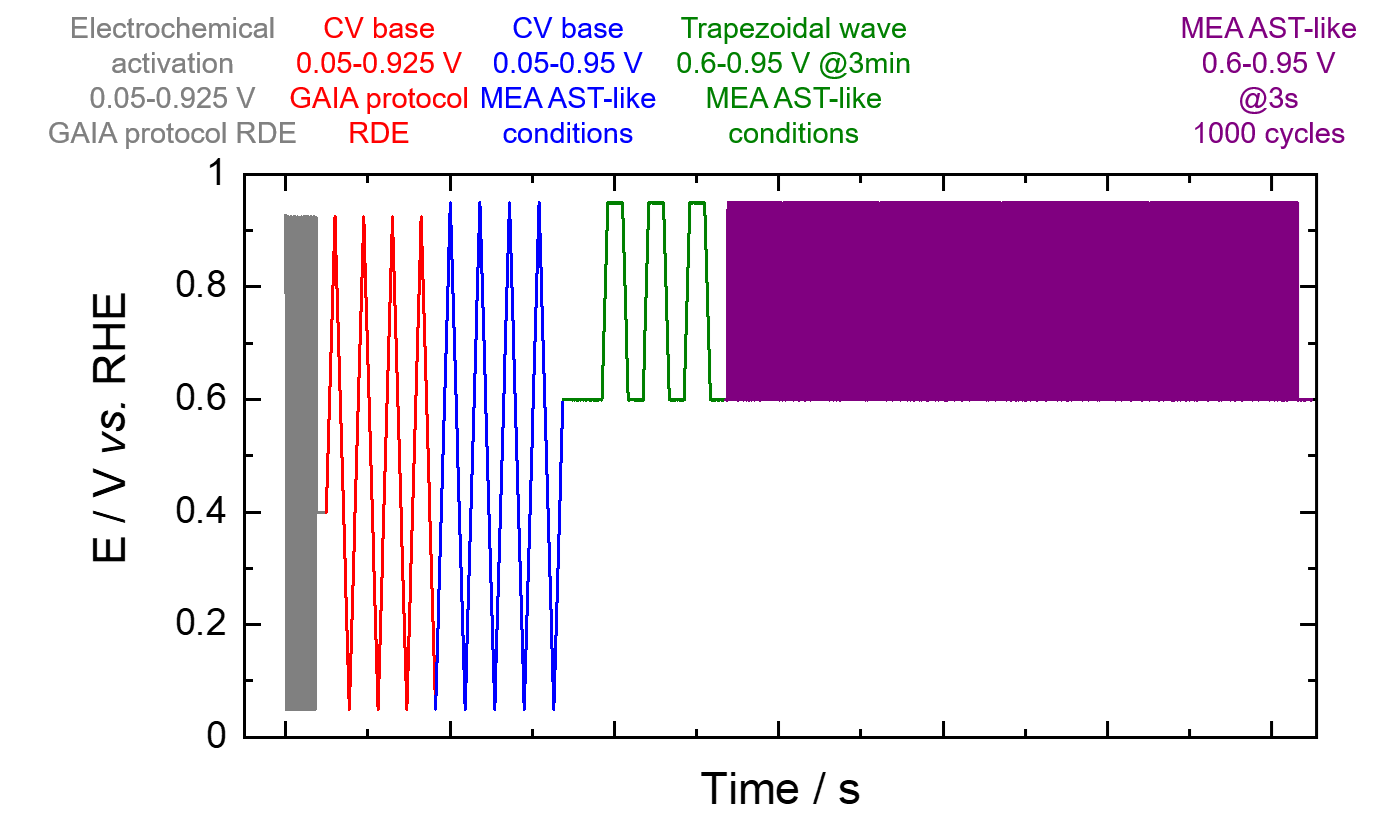


Figure S14: Electrochemical protocol of *online* ICP-MS measurement.


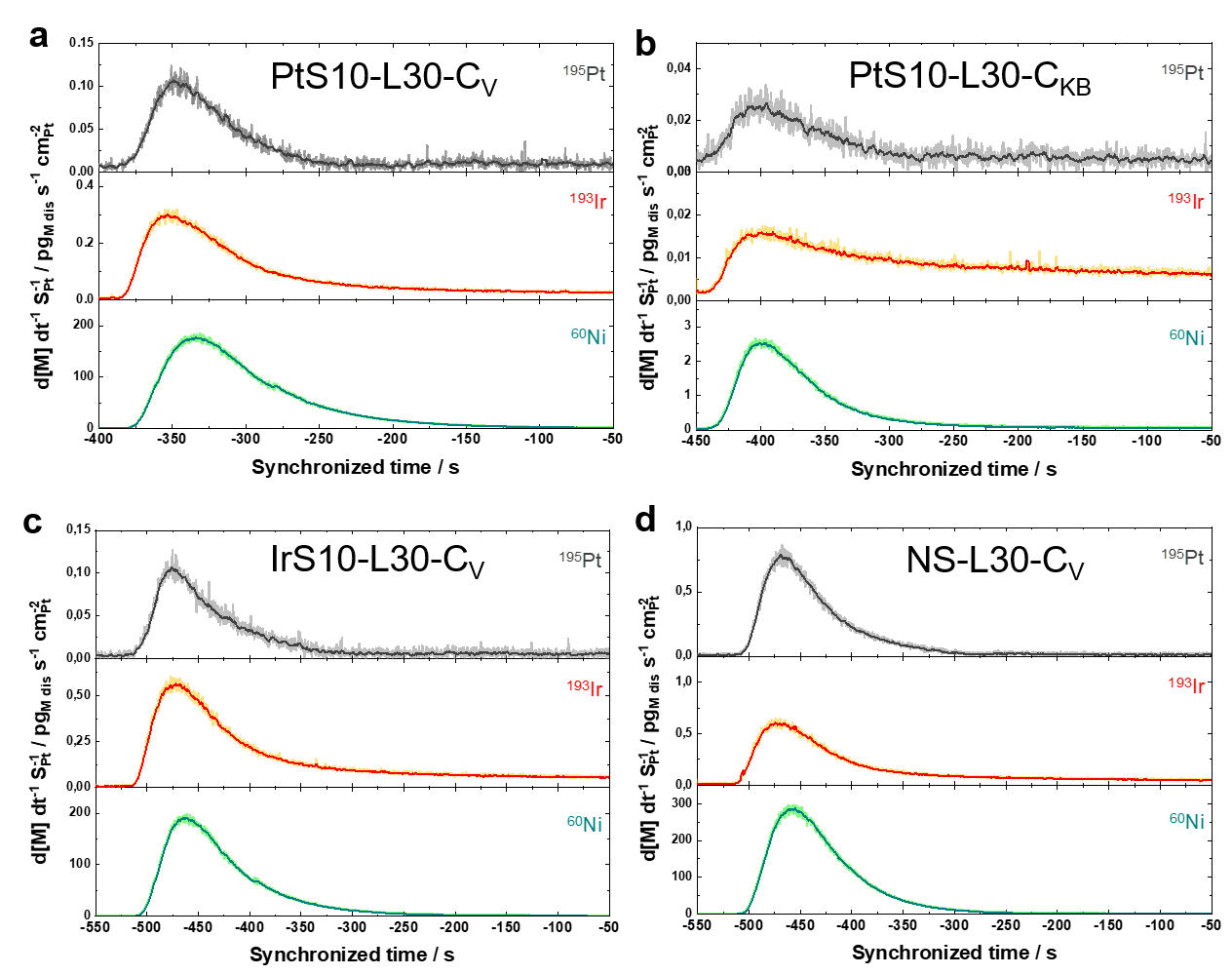


Figure S15: *Online* ICP-MS signal of Pt (black, top), Ir (red, middle) and Ni (green, bottom) during the first electrode contact with the electrolyte of a) PtS10-L30-C_V_, b) PtS10-L30-C_KB_, c) IrS10-L30-C_V_ and d) NS-L30-C_V_.


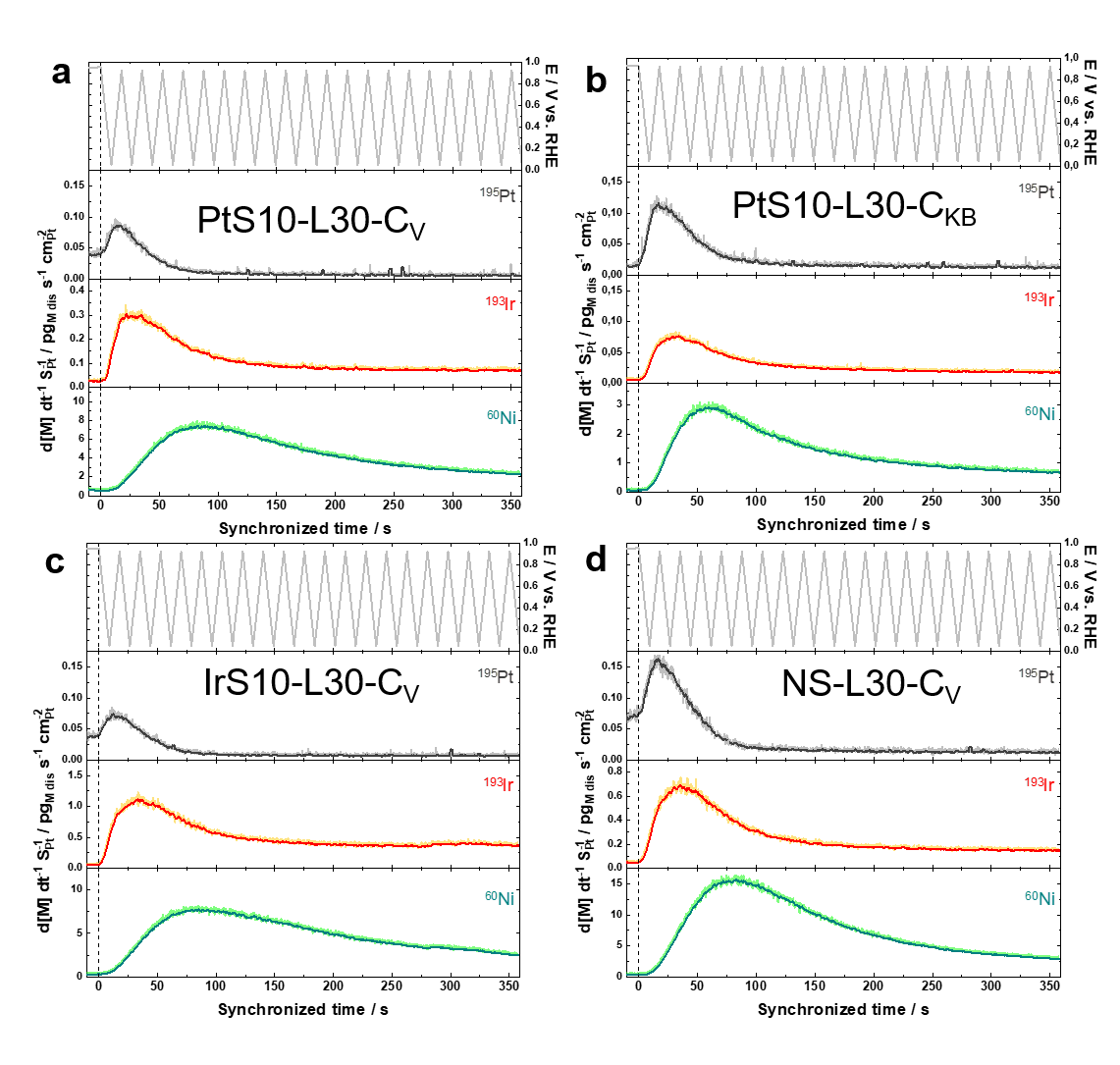


Figure S16: *Online* ICP-MS signal of Pt (black, top), Ir (red, middle) and Ni (green, bottom) during activation step of a) PtS10-L30-C_V_, b) PtS10-L30-C_KB_, c) IrS10-L30-C_V_ and d) NS-L30-C_V_.


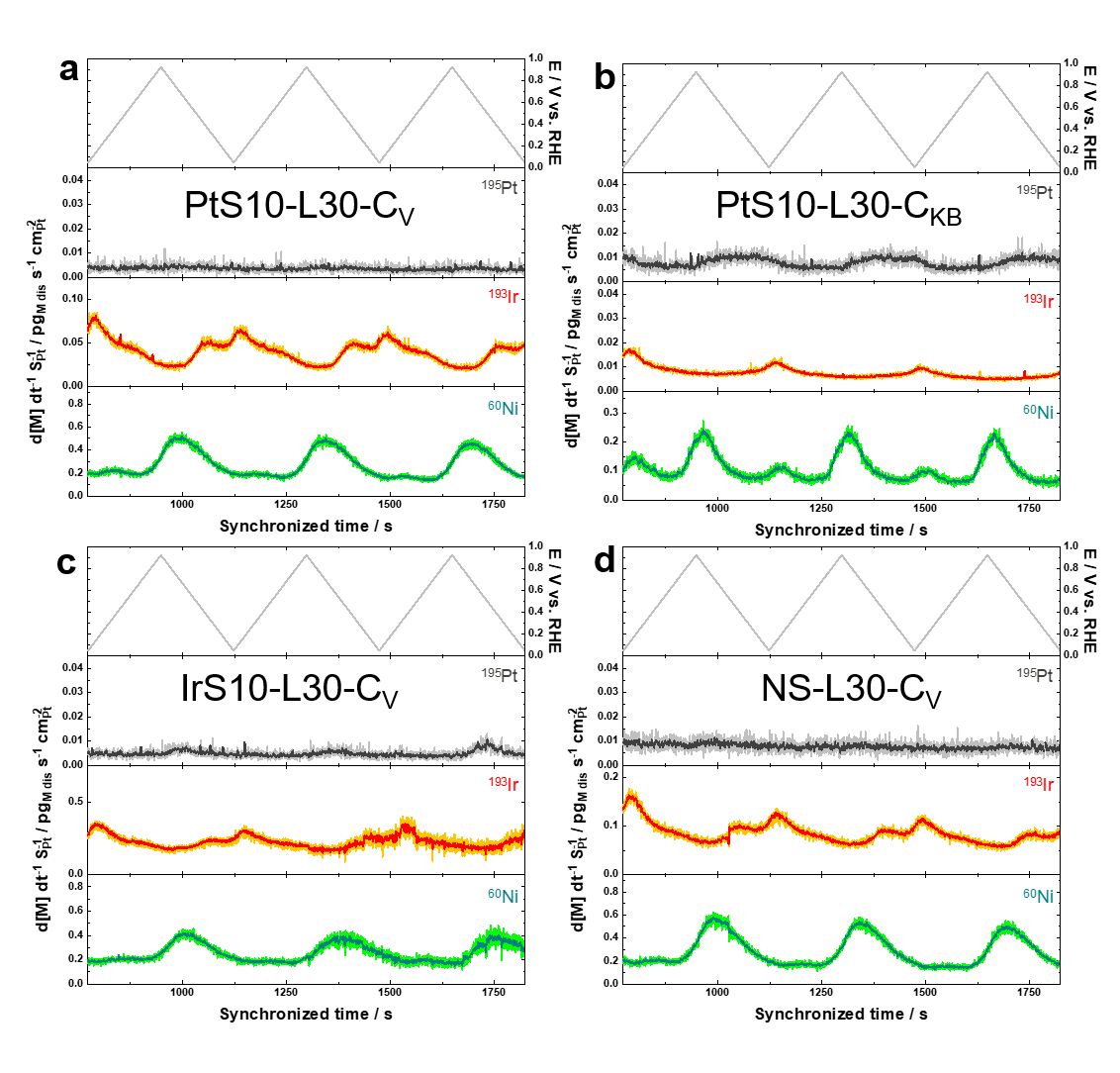


Figure S17: *Online* ICP-MS signal of Pt (black, top), Ir (red, middle) and Ni (green, bottom) during CV based GAIA RDE protocol of a) PtS10-L30-C_V_, b) PtS10-L30-C_KB_, c) IrS10-L30-C_V_ and d) NS-L30-C_V_.


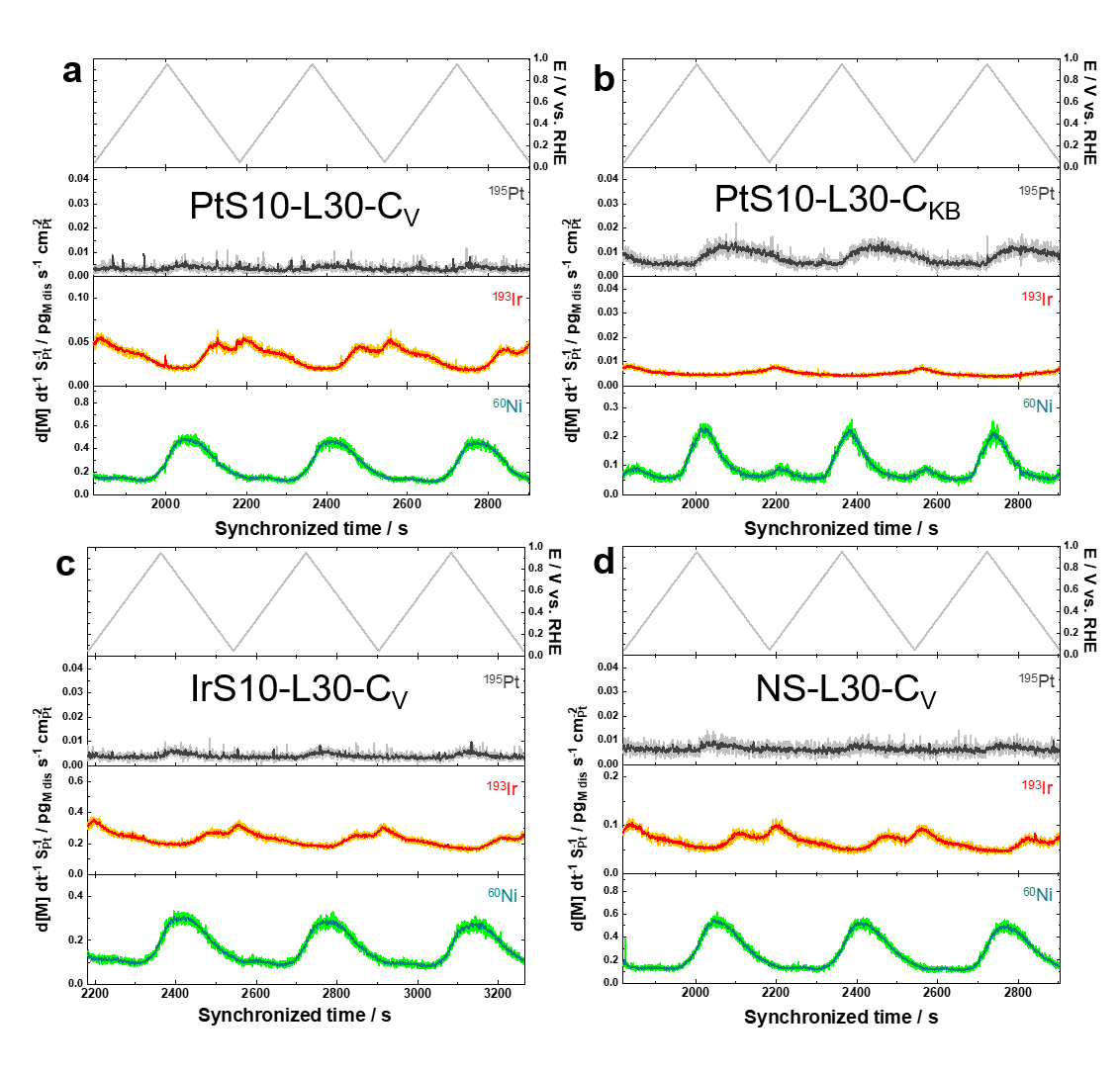


Figure S18: *Online* ICP-MS signal of Pt (black, top), Ir (red, middle) and Ni (green, bottom) during CV based MEA AST-like protocol of a) PtS10-L30-C_V_, b) PtS10-L30-C_KB_, c) IrS10-L30-C_V_ and d) NS-L30-C_V_.


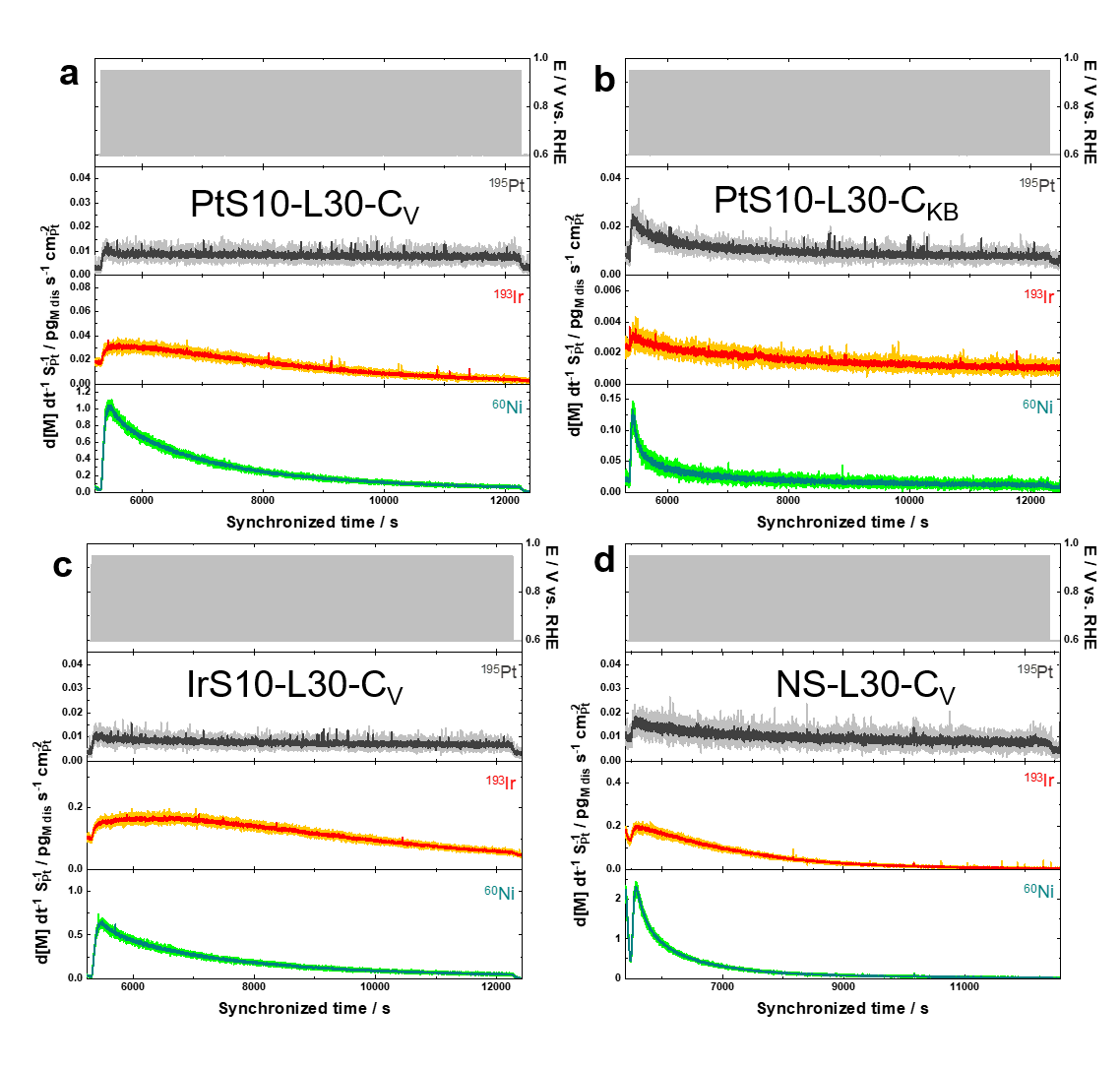


Figure S19: *Online* ICP-MS signal of Pt (black, top), Ir (red, middle) and Ni (green, bottom) during MEA AST-like protocol of a) PtS10-L30-C_V_, b) PtS10-L30-C_KB_, c) IrS10-L30-C_V_ and d) NS-L30-C_V_.


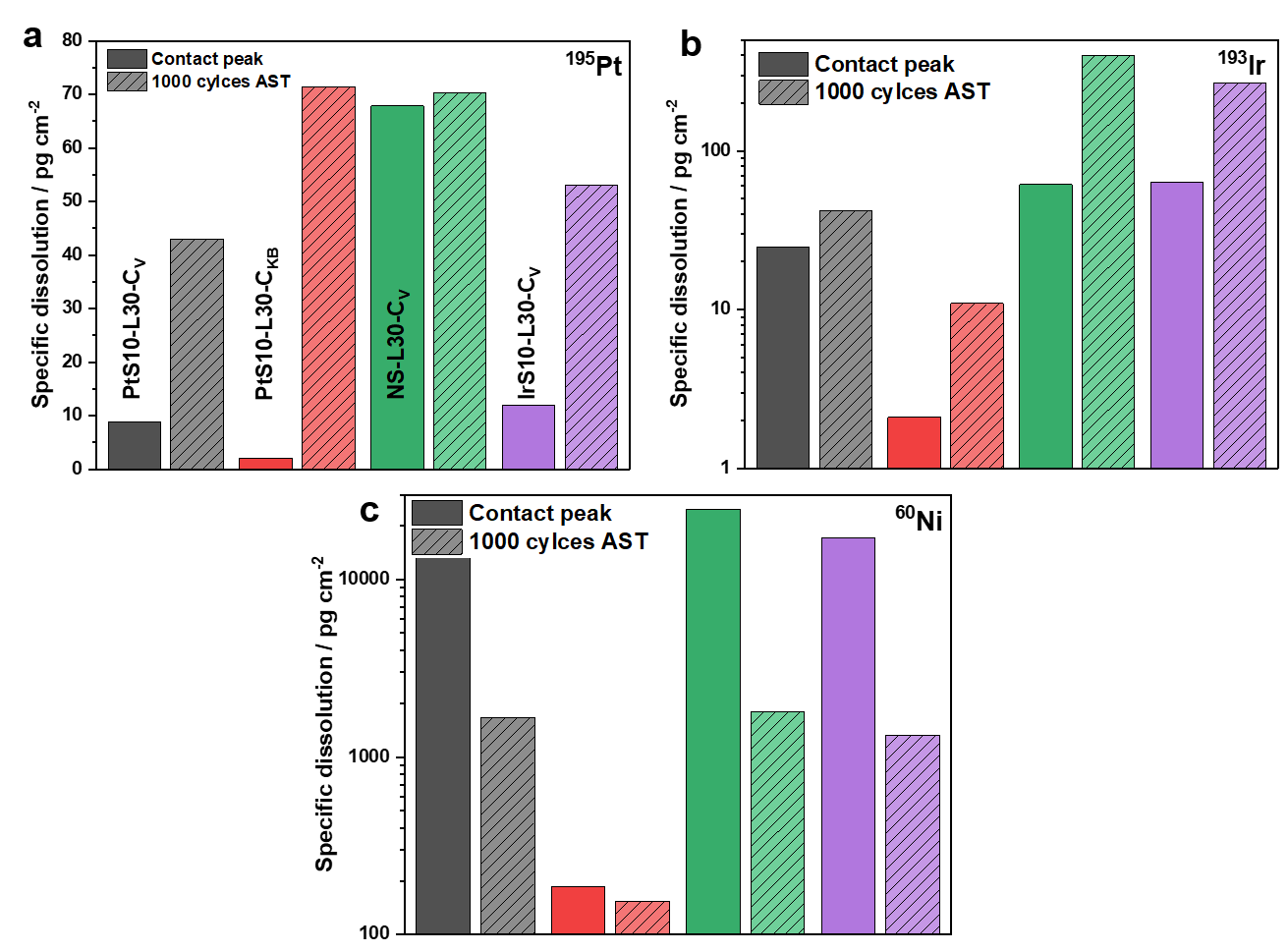


Figure S20: Specific metal dissolution of a) Pt, b) Ir and c) Ni of four samples integrated signal during contact peak (dark) and 1000 cycles AST (light).

**References**

1. E. Hornberger, T. Merzdorf, H. Schmies, J. Hubner, M. Klingenhof, U. Gernert, M. Kroschel, B. Anke, M. Lerch, J. Schmidt, A. Thomas, R. Chattot, I. Martens, J. Drnec and P. Strasser, *ACS Appl Mater Interfaces*, 2022, **14**, 18420-18430, 10.1021/acsami.2c00762.

2. U. S. D. o. Energy, Multi-Year Research, Development, and Demonstration Plan - Section 3.4 Fuel Cells, (accessed 09.03.2024).
